# Targeting of *Leishmania* proteins by β-Lapachone derivatives reveals a promising multitarget candidate

**DOI:** 10.1007/s00203-026-04989-1

**Published:** 2026-06-06

**Authors:** Laércio Mariano-Fernandes, Jacilene Silva, Matheus Nunes da Rocha, Márcia Machado Marinho, Emmanuel Silva Marinho

**Affiliations:** 1 Postgraduate Program in Veterinary Sciences, State University of Ceará, Fortaleza, CE Brazil; 2 Postgraduate Program in Biological Chemistry, Regional University of Cariri, Crato, CE Brazil; 3 Postgraduate Program in Natural Sciences – PPGCN, State University of Ceará, Fortaleza, Brazil; 4 Bioprospecting and Natural Resource Monitoring Laboratory, State University of Ceará, Av. Dr. Silas Munguba, 1700 - Itaperi Campus, Fortaleza, CEP: 60.714.903 CE Brazil

**Keywords:** Dihydroorotate dehydrogenase, N-myristoyltransferase, Cathepsin B, Molecular docking, Conformational dynamics, ADMET

## Abstract

Leishmaniasis represents one of the major global public health problems. Canine leishmaniasis plays a significant role in perpetuating this condition, due to the limited therapeutic options available, which are costly, require prolonged treatment regimens, and are associated with the growing emergence of drug resistance. In this context, β-lapachone, a bioactive naphthoquinone derived from lapachol, has emerged as a potential therapeutic alternative due to its pharmacological properties, including antiparasitic activity, which characterizes it as a promising alternative for the development of new antileishmanial agents. In this study, a group of β-lapachone derivatives was evaluated using an integrated computational approach to investigate their antileishmanial potential and pharmacokinetic profile. Molecular docking simulations indicated greater selectivity of the compounds for *Leishmania *spp. targets compared to currently used drugs. Among the derivatives analyzed, compound LPI3 stood out as the lead compound, exhibiting a promising multitarget profile. Molecular dynamics analyses based on normal mode analysis revealed high conformational stability of the protein-ligand complexes involving LPI3, similar to that observed for the reference drugs. The physicochemical properties, as well as predictions of pharmacokinetic descriptors related to absorption, distribution, metabolism, excretion, and toxicity, indicated a favorable profile for the lead compound, consistent with drug-likeness and safety criteria. Therefore, the findings identified LPI3 as a potential candidate for future experimental evaluations in the treatment of leishmaniasis.

**Graphical abstract:**

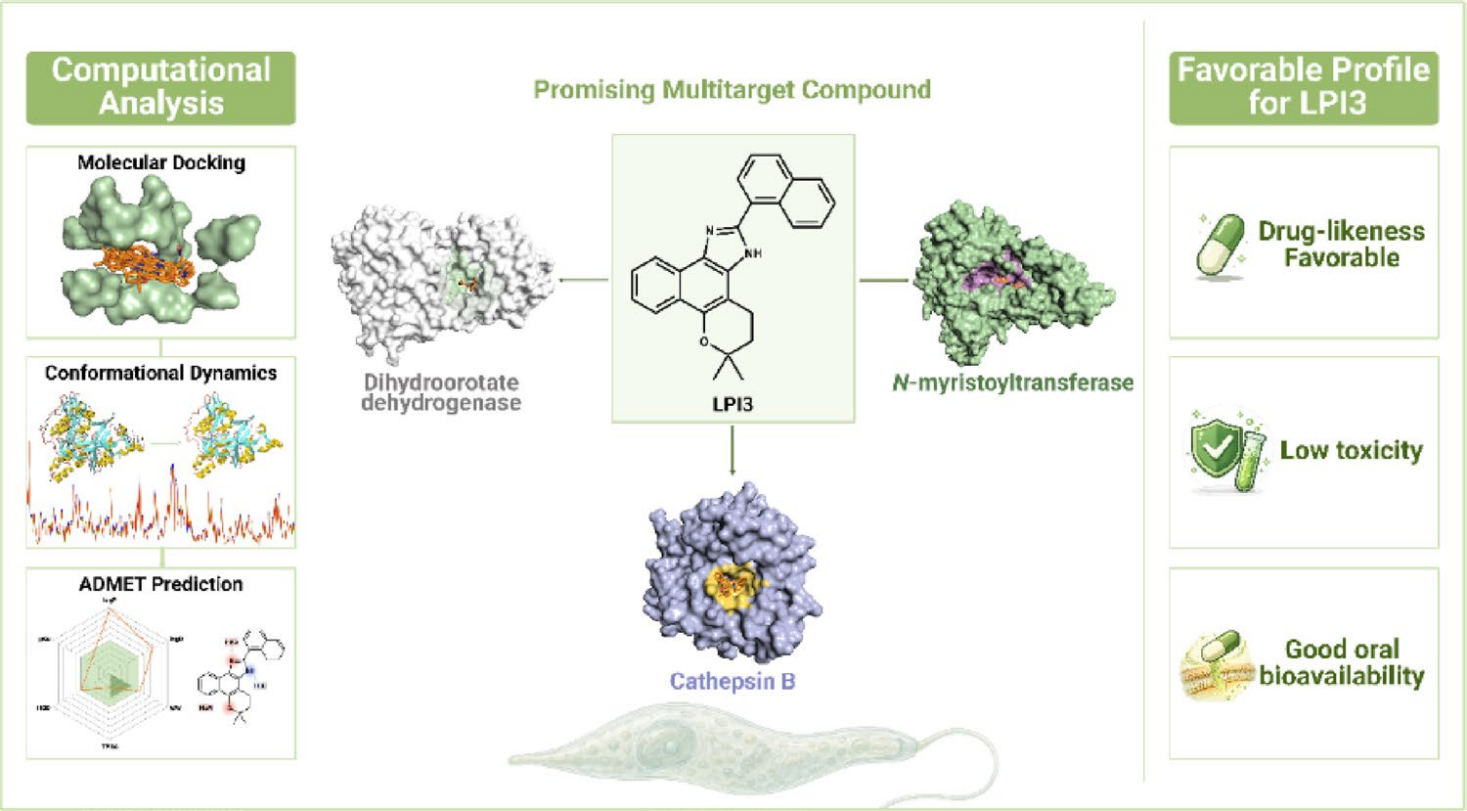

**Supplementary Information:**

The online version contains supplementary material available at 10.1007/s00203-026-04989-1.

## Introduction

Leishmaniasis is a group of parasitic diseases caused by intracellular flagellated protozoa belonging to the genus *Leishmania* (Akhoundi et al. 2016). This condition is widespread in tropical and subtropical regions, occurring in 99 countries across Africa, the Americas, Asia, and Europe, directly affecting both animals and humans (Maia et al. 2023; Jain et al. 2025). It is estimated that between 0.7 and 1.1 million people are infected annually worldwide, and that a smaller fraction of these cases progress to severe forms and eventual death (WHO 2023). As a result, leishmaniasis is considered one of the major neglected tropical diseases, constituting one of the world’s greatest public health problems, with a rising infection rate and a strong association with poverty (Alvar et al. 2006; Chen et al. 2025a).

In this context, canine leishmaniasis (CanL) plays a key role in the rise of human infections, given that dogs are the primary reservoir for *Leishmania *spp., that a proportion of infected dogs may remain asymptomatic, and that these animals share the same environment with humans (Ribeiro et al. 2018; Priolo et al. 2024). Regarding epidemiology, CanL exhibits high prevalence rates in different regions of the world, potentially exceeding 70% in endemic areas (Vilas-Boas et al. 2024). However, the prevalence of infection in dogs may vary depending on the population studied, the location, and the diagnostic method used, with average values ranging from 11.7% to 17.4% (Vilas-Boas et al. 2024; Priolo et al. 2024; Plesko et al. 2025).

The life cycle of *Leishmania *spp. involves a mammalian host and a sandfly vector (Clos et al. 2022). Female sandflies of the genera *Phlebotomus* and *Lutzomyia* become infected during a blood meal from a host carrying leishmaniasis (Esch and Petersen 2013). Subsequently, during a new blood meal, the infected sandfly inoculates infective forms of the parasite, which are phagocytosed by neutrophils and macrophages of the mammalian host (Lestinova et al. 2017). Once internalized within these cells, these protozoa employ multiple mechanisms of immune evasion and modulation to promote their survival and proliferation during infection (Carneiro et al. 2021).

Clinically, CanL, associated primarily with *L. infantum* and *L. donovani*, can manifest as tegumentary (TL) − subdivided into cutaneous, diffuse, or mucocutaneous forms − and visceral (VL) forms, defined mainly by the parasite’s location in the tissues, the types of phagocytic cells invaded, and the severity of the clinical presentation (Alvar et al. 2012). The tegumentary form affects the skin and mucous membranes, while the visceral form, considered the most pathogenic form of the disease, compromises cells of the mononuclear phagocytic system in various organs and can be fatal if left untreated. Human TL is most frequently associated with the species *L. major*, *L. tropica*, and *L. braziliensis*, while VL and CanL are caused by the species *L. infantum* and *L. donovani* (Reithinger et al. 2007; Sasani et al. 2016; Pareyn et al. 2025).

With regard to treatment, the options available in veterinary medicine are limited and are typically aimed at alleviating clinical signs rather than completely eliminating the parasite, in addition to the implementation of public health control measures, such as the euthanasia of seropositive animals, highlighting the complexity and impact of this disease on animal health (Sarquis et al. 2025; Saridomichelakis et al. 2025). Thus, CanL represents a significant clinical problem and a critical factor in sustaining zoonotic transmission. In this context, various drugs with antileishmanial activity have been used to treat this condition, including pentavalent antimonials, amphotericin B (amphoB), miltefosine (MTF), and paromomycin (Haldar et al. 2011; Palić et al. 2022; Sheikh et al. 2024). Although they have proven clinical efficacy, these medications exhibit limited therapeutic effects, high costs, the need for prolonged treatment regimens, the development of drug resistance resulting from this extensive use, and the occurrence of significant adverse effects, such as cardiac and hepatic abnormalities, high toxicity rates, and teratogenicity (Laniado-Laborín and Cabrales-Vargas 2009; Oliveira et al. 2011; Abirami et al. 2023). Therefore, these limitations highlight the need for new, safer, more effective, and more selective therapeutic approaches for the treatment of leishmaniasis.

Thus, the selection of molecular targets is an indispensable process for the development of new antileishmanial drugs, in order to identify essential proteins associated with the protozoan’s ability to replicate, its pathogenic potential, and its survival, the inhibition of which can compromise the development, viability, and establishment of the infection (Jain and Jain 2018; Challapa-Mamani et al. 2023). Among these targets, dihydroorotate dehydrogenase (DHODH) stands out, a flavoprotein enzyme involved in the fourth step of the *de novo* pyrimidine biosynthesis pathway, which is essential for replication and cell division processes (Feliciano et al. 2006; Cordeiro et al. 2012). Similarly, *N*-myristoyltransferase (NMT) is a monomeric enzyme essential for the survival of *Leishmania* parasites, responsible for catalyzing *N*-myristoylation, a post-translational modification involved in vesicular protein transport, signal transduction, and the regulation of protein complex formation and stability (Corpas-Lopez et al. 2019; Khalil et al. 2019). Furthermore, cathepsin B (CatB), present in the trypanosomatids *Leishmania *spp. and *Trypanosoma *spp., is a cysteine protease localized in lysosomal compartments, which acts as an important pathogenicity factor in the process of tissue invasion (Xie et al. 2023).

In addition, the growing demand for innovative and effective treatments to combat leishmaniasis has spurred research into natural products, due to their structural diversity and broad-spectrum bioactivity (Gervazoni et al. 2020). Among these compounds, β-lapachone is noteworthy, an ortho-naphthoquinone derived from lapachol, recognized for its significant pharmacological potential and the possibility of structural modifications that can enhance its selectivity and therapeutic efficacy (Gómez Castellanos et al. 2009). From a physicochemical perspective, it is a small lipophilic molecule with low water solubility, remaining unionized at physiological pH, characteristics that may influence its bioavailability and interaction with biological targets (Bermejo et al. 2017; Kim et al. 2018). Regarding its bioactivity, β-lapachone exhibits antineoplastic, anti-inflammatory, antioxidant, neuroprotective, nephroprotective, and wound-healing effects, standing out as a valuable therapeutic agent with potential against *Leishmania *spp. (Gomes et al. 2021; Ramos-Milaré et al. 2022).

At the same time, computational methods have established themselves as effective strategic tools in the discovery and development of new antiparasitic drugs, as they allow for a detailed investigation of the interactions and stability of complexes formed by different ligands and target proteins of the protozoan, in addition to predicting the pharmacokinetic, pharmacodynamic, and toxicological properties of the compound, thereby reducing costs, experimental time, and the use of biological models (Kitchen et al. 2004; Meng et al. 2011; Zhong 2017). In light of this, this study aims to evaluate, through an integrated approach using computational methods, the pharmacokinetic and pharmacodynamic profiles and molecular interactions of β-lapachone derivatives with essential protein targets of *Leishmania *spp. to estimate their antileishmanial potential.

## Materials and methods

### Methodological design

**Fig. 1:**
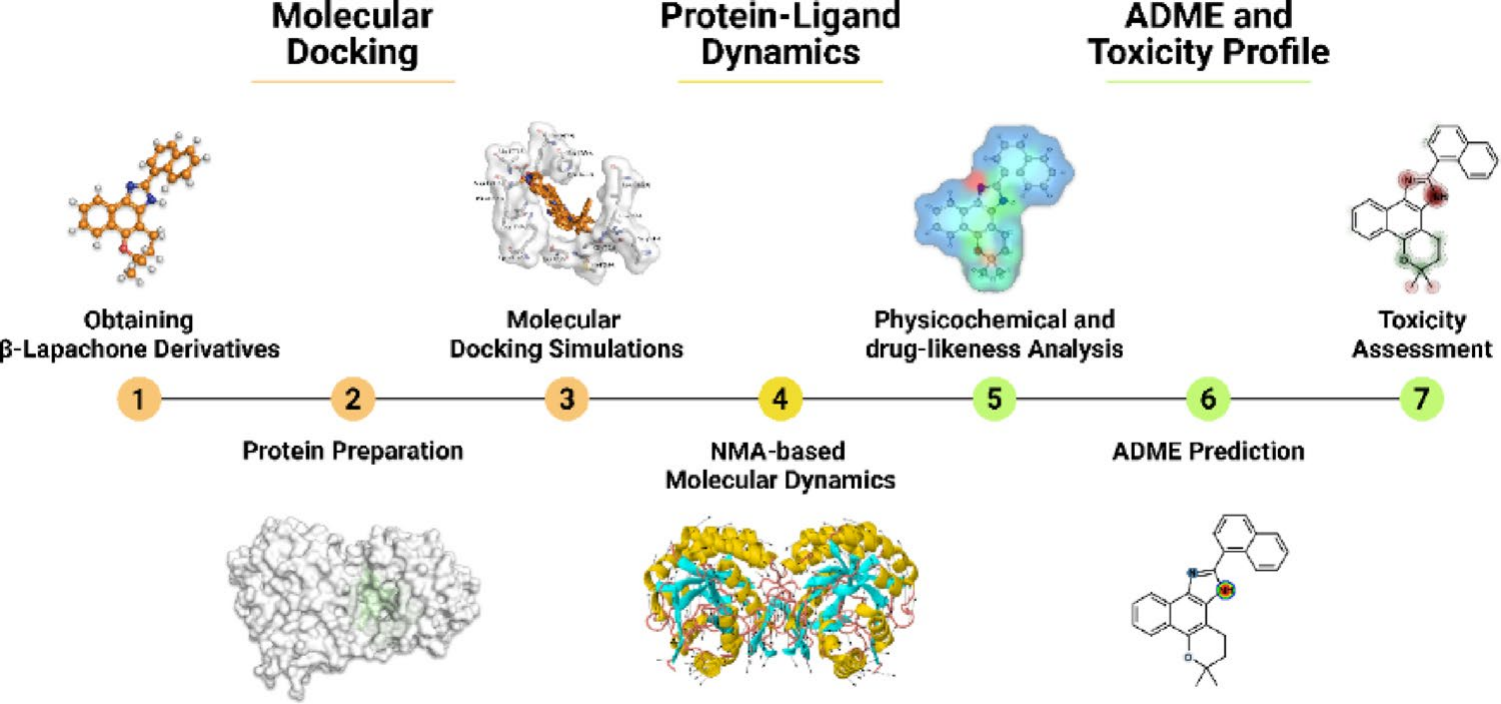
Flowchart of the integrated computational approach for evaluating the antileishmanial potential of β-lapachone derivatives as inhibitors of molecular targets of *Leishmania spp*

Initially, the structures of the molecular targets were validated and structurally characterized. Then, the proteins and ligands were prepared and subjected to molecular docking simulations, enabling the prediction of binding modes and a detailed analysis of the interactions. Subsequently, the selected lead compound underwent conformational dynamics analysis to assess the stability of the complex, as well as structural flexibility and deformability via Normal Mode Analysis (NMA). Finally, the compound was evaluated for its pharmacokinetic and toxicological properties in silico to estimate its absorption, distribution, metabolism, excretion, and toxicity (ADMET) profile. This methodological approach was employed to identify compounds with potential antileishmanial activity (Fig. 1).

### Rendering and structural optimization of β-Lapachone derivatives

**Fig. 2:**
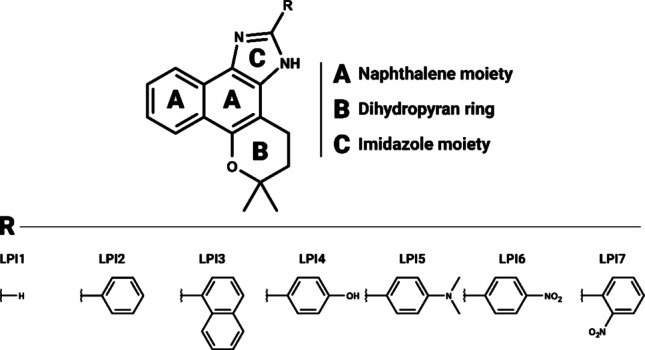
β-lapachone derivatives selected for structure-based virtual screening. Ligands LPI1 − LPI7 were ranked according to the characterization reported in (Santos et al. 2023)

The study by Santos et al. (Santos et al. 2023) was used to obtain the two-dimensional (2D) structures of the β-lapachone derivatives (Fig. 2). All structures were plotted and rendered using the academic-license software MarvinSketch (https://chemaxon.com/marvin). Then, the structures underwent three-dimensional (3D) modeling and structural optimization using Avogadro 1.2.0 software (http://avogadro.cc/) (De Oliveira et al. 2025). Subsequently, semi-empirical calculations were performed in MOPAC software (https://openmopac.net/about/), using the PM7 method and the Merck Molecular Force Field 94 (MMFF94) to achieve a lower energy state.

### Protein structural validation, pocket prediction, and preparation

To investigate the mechanism of action of the compounds, 3D structures of the molecular targets were obtained from the Protein Data Bank (PDB) repository (https://www.rcsb.org/). The structure of the DHODH enzyme (PDB ID: 3MHU), identified as ‘Crystal structure of dihydroorotate dehydrogenase from *Leishmania major* in complex with 5-Nitroorotic acid’, was deposited with a resolution of 1.85 Å, determined by X-ray diffraction (Cheleski et al. 2010). The enzymatic structure of NMT (PDB ID: 2WUU), identified as ‘Structure of *N*-myristoyltransferase from *L. donovani*’, was crystallized by X-ray diffraction at a resolution of 1.42 Å (Brannigan et al. 2010). Regarding CatB (PDB ID: 3HHI), described as ‘Crystal Structure of Cathepsin B from *T. brucei* in complex with CA074’, it was obtained by X-ray diffraction at a resolution of 1.60 Å (Kerr et al. 2010).

To verify and validate the protein models, the PDB file corresponding to each structure was subjected to conformation analysis using the Ramachandran plot on the Ramplot server (https://www.ramplot.in/index.php). This tool was configured to assess the stereochemical quality of the proteins by evaluating the geometry generated by the dihedral angles of the bonds for each residue. Subsequently, the same file was analyzed using the DoGSite3 − Proteins Plus Tool (https://proteins.plus/) to estimate potential binding pockets, including values for their volumes, surface areas, and depths.

Protein preparation was performed using AutoDockTools software, involving the removal of non-essential residues, the addition of polar hydrogen atoms, and the inclusion of Kollman and Gasteiger charges (Morris et al. 2009; Gomes et al. 2023). The dimensions of the grid boxes were defined to fully encompass the catalytic sites described for each molecular target, including adjacent regions that may be relevant for ligand-receptor stabilization. Consequently, a semi-blind approach was adopted, allowing for greater conformational exploration of the environment surrounding the active site without unduly restricting the conformational search. For the DHODH enzyme structure, the flavin mononucleotide (FMN) residues were retained. The grid box for docking was centered at −5.503, 53.797, and 14.183 for the *x*, *y*, and *z* axes, with sizes of 86 Å, 68 Å, and 97 Å. For NMT, the grid box was centered at −10.15, −24.43, and 21.73 on the *x*, *y*, and *z* axes, with sizes of 94 Å, 112 Å, and 105 Å, respectively. As for CatB, the centering coordinates were 6.854, 1.555, and 35.375 for the *x*, *y*, and *z* axes, with respective sizes of 114 Å, 91 Å, and 100 Å.

### Molecular docking simulations

All docking simulations were performed using AutoDockVina software, configured to run 50 independent simulations, generating 20 conformational possibilities for each ligand (Trott and Olson 2010; Marinho et al. 2020). The protein structures were kept rigid during the simulations, while all rotatable bonds of the ligands were considered flexible, due to the high computational demands associated with flexible docking, especially given the number of targets and compounds investigated. Furthermore, as criteria for filtering the best ligand conformation, values below 2.0 Å for Root Mean Square Deviation (RMSD), indicating low conformational variation, and affinity energies (E_A_) below −6.0 kcal/mol were adopted (Yusuf et al. 2008; Shityakov and Foerster 2014). The standard scoring function of AutoDock Vina was retained due to its widespread use and validation in molecular docking studies, demonstrating robust performance in predicting binding poses and relative affinities. The simulations were validated using the redocking technique. The data obtained were compared with the reference drugs MTF (PubChem CID: 3599) and AmphoB (PubChem CID: 5280965) (Marinho et al. 2024; Da Silva et al. 2026).

### NMA-based conformational dynamics analysis

NMA-based conformational dynamics analysis were performed using the iMODS server (https://imods.iqf.csic.es/) to evaluate the flexibility, deformability, and conformational stability of the protein-ligand complexes obtained from molecular docking (López-Blanco et al. 2014). Initially, the PDB-format files of the protein−ligand complexes, including the lead compound, reference drugs, and co-crystallized inhibitors corresponding to each molecular target, were submitted to the server for analysis. The characterization of structural dynamics was performed based on parameters derived from NMA. The Root Mean Square Fluctuation (RMSF) provided information on flexibility at the residue level, while deformability was used to identify regions with greater intrinsic mobility and potential for conformational adaptation. Finally, NMA-based morphing analysis, given by the Root Mean Square Deviation (RMSD) of alpha carbon atoms (Cα) across iterations, was performed to measure conformational transitions between different structural states. In this step, the complexes obtained by molecular docking were considered as the first component, while the corresponding experimental structures, obtained from the Protein Data Bank, were used as references, with the PDB codes 3TJX (DHODH), 4CGL (NMT), and 3MOR (CatB) (Koopmann et al. 2012; De Souza et al. 2012; Brannigan et al. 2014).

### MPO analysis and ADMET prediction

#### Physicochemical properties and drug-likeness criteira

The analysis of the physicochemical properties and Multiparameter Optimization (MPO) was performed according to Da Rocha et al. 2024. Initially, MarvinSketch software was used to calculate the ionization constant (pKa), and based on this, the distribution of the molecule’s chemical groups was applied to estimate the oral bioavailability score (ABS) on the SwissADME server (http://www.swissadme.ch/). On the same server, information regarding physicochemical properties was obtained, including molecular weight (MW), number of aromatic rings (AR), number of rotatable bonds (nRot), number of hydrogen bond acceptors (HBA) and donors (HBD), topological polar surface area (TPSA), partition coefficient (logP), and distribution coefficient (logD).

Additionally, the quantitative estimate of drug-like properties (QED) was calculated, as proposed by Bickerton et al. 2012– Eq. 1:

1 $$\:QED={exp}\left(\frac{1}{2}{\sum\:}_{i=1}^{n}{lnd}_{i}\right)$$

this descriptor is based on a desirability function (*d*) applied to multiple structural attributes (*i*) (*n* = 8), including MW ≤ 500 g/mol, logP ≤ 5, HBA ≤ 10, and HBD ≤ 5, in accordance with Lipinski’s Rule; TPSA ≤ 140 Å^2^, nRot ≤ 10, according to Veber’s criteria; in addition to the number of aromatic rings (AR ≤ 3) and the presence of Brenk’s structural alerts (Veber et al. 2002; Lipinski 2004; Brenk et al. 2008; Ritchie et al. 2011). The sum of these factors results in a score ranging from 0.0 (lowest desirability) to 1.0 (highest desirability).

The biopharmaceutical classification system based on MPO developed by Pfizer, Inc., as described by Wager et al. 2010b; was also employed to quantitatively estimate the ADMET feasibility of the compounds, as expressed in Eq. 2:

2 $$\:D=\:{\sum\:}_{i=1}^{n}{w}_{k}{T}_{k}\left({x}_{k}^{0}\right)$$

In this model, the desirability function (*D*) is defined as the weighted sum (*w*) of individual structural attributes (*k*), whose values *x*_*k*_ are evaluated relative to ideal limits (*T*_*k*_). The parameters considered include MW ≤ 360 g/mol, 40 Å² < TPSA ≤ 90 Å², HBD ≤ 1, most basic pKa ≤ 8, logP ≤ 3, logD ≤ 2 (*n* = 6). The resulting MPO score ranges from 0.0 to 6.0, where values of MPO ≥ 3 − 4 indicate compounds with a balanced pharmacokinetic profile and properties compatible with promising drug candidates, as described by Johnson et al. 2009; Wager et al. 2010a.

#### Evaluation of ADMET properties

The lead compound was evaluated using various server tools for predicting pharmacokinetic properties, including SwissADME, pkCSM (https://biosig.lab.uq.edu.au/pkcsm/), and ADMETlab 3.0 (https://admetlab3.scbdd.com/). For absorption, the descriptors evaluated include water solubility, permeability in Caco-2 cells, human intestinal absorption (HIA), skin permeability, and action as a substrate or inhibitor of P-glycoprotein (Pgp) Regarding distribution, the estimated descriptors include steady-state volume of distribution (VDss) and plasma free fraction (Fu), as well as permeability across the blood-brain barrier (BBB) and into the central nervous system (CNS).

#### Site of metabolism prediction

Metabolism-wise, a predictive analysis of the compound’s biotransformation mediated by the major cytochrome P450 (CYP450) isoenzymes was conducted to elucidate possible metabolic elimination pathways, including the CYP1A2, CYP2C9, CYP2C19, CYP2D6, and CYP3A4 isoforms. In this regard, the prediction made by the aforementioned servers was confirmed by the model available on the XenoSite server (https://xenosite.org/). In addition, the prediction of the Phase I site of metabolism (SOM) for the lead compound was performed using the academic tool FAME3R (https://nerdd.univie.ac.at/fame3r), while the identification of possible metabolites generated during biotransformation was verified using the GLORY tool (https://nerdd.univie.ac.at/glory). Both resources are available on the Next-generation E-Resource for Drug Discovery (NERRD) server (https://nerdd.univie.ac.at/). The XenoSite server was also used to estimate the formation of likely Phase II metabolites.

For the prediction of excretion, the descriptors evaluated include total clearance and the compound’s action as a substrate for the OCT2 (Organic Cation Transporter 2) protein, which plays a role in the renal elimination of drugs and endogenous compounds (Ailabouni and Prasad 2025).

#### Toxicity prediction

To estimate the toxic response, the evaluated descriptors include the maximum tolerated dose in humans, as well as chronic (LOAEL) and acute (LD_50_) oral toxicity in experimental models. The probability of mutagenicity (AMES) and hepatotoxicity was also estimated. The cardiotoxicity assessment included the prediction of the potential for inhibition of the hERG (human ether-a-go-go-related gene) channel, an important marker of arrhythmia risk. The analysis was conducted using the ADMET Prediction Service – LMC (http://qsar.chem.msu.ru/admet/) and the pre-hERG 5.0 tool (https://predherg.labmol.com.br/), which estimate the probability of this channel being blocked by the compound. Finally, the prediction of acute toxicity via inhalation and oral ingestion, as well as eye and skin irritation and corrosion, was performed using the STopTox server (https://stoptox.mml.unc.edu/).

## Results

### Protein structural validation and pocket prediction

**Fig. 3:**
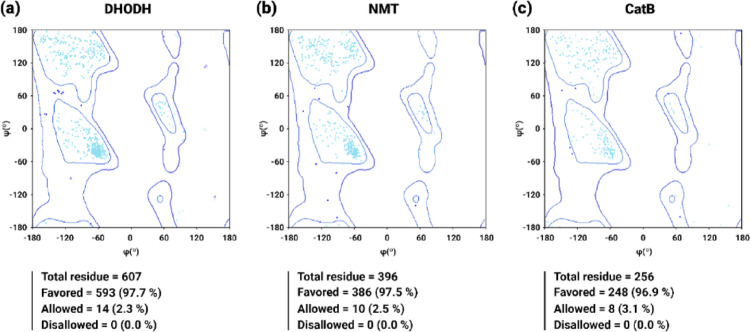
Structural validation of the crystallized structures of **a** dihydroorotate dehydrogenase (DHODH), **b** *N*-myristoyltransferase (NMT), and **c** cathepsin B (CatB) using the Ramachandran plot. Cyan, blue, and red points represent torsion angles of favored, allowed, and forbidden regions, respectively

The structural validation of the molecular targets DHODH, NMT, and CatB, using the Ramachandran model, is shown in Fig. 3. Analysis of the distribution of residues by energy regions revealed excellent stereochemical quality of the proteins, characterized by the absence of residues in disallowed regions and a high concentration in energetically favored regions. In this case, DHODH (Fig. 3a) had 97.7% of its residues distributed in favorable regions, while NMT (Fig. 3b) and CatB (Fig. 3c) had 97.5% and 96.9%, respectively.

**Fig. 4:**
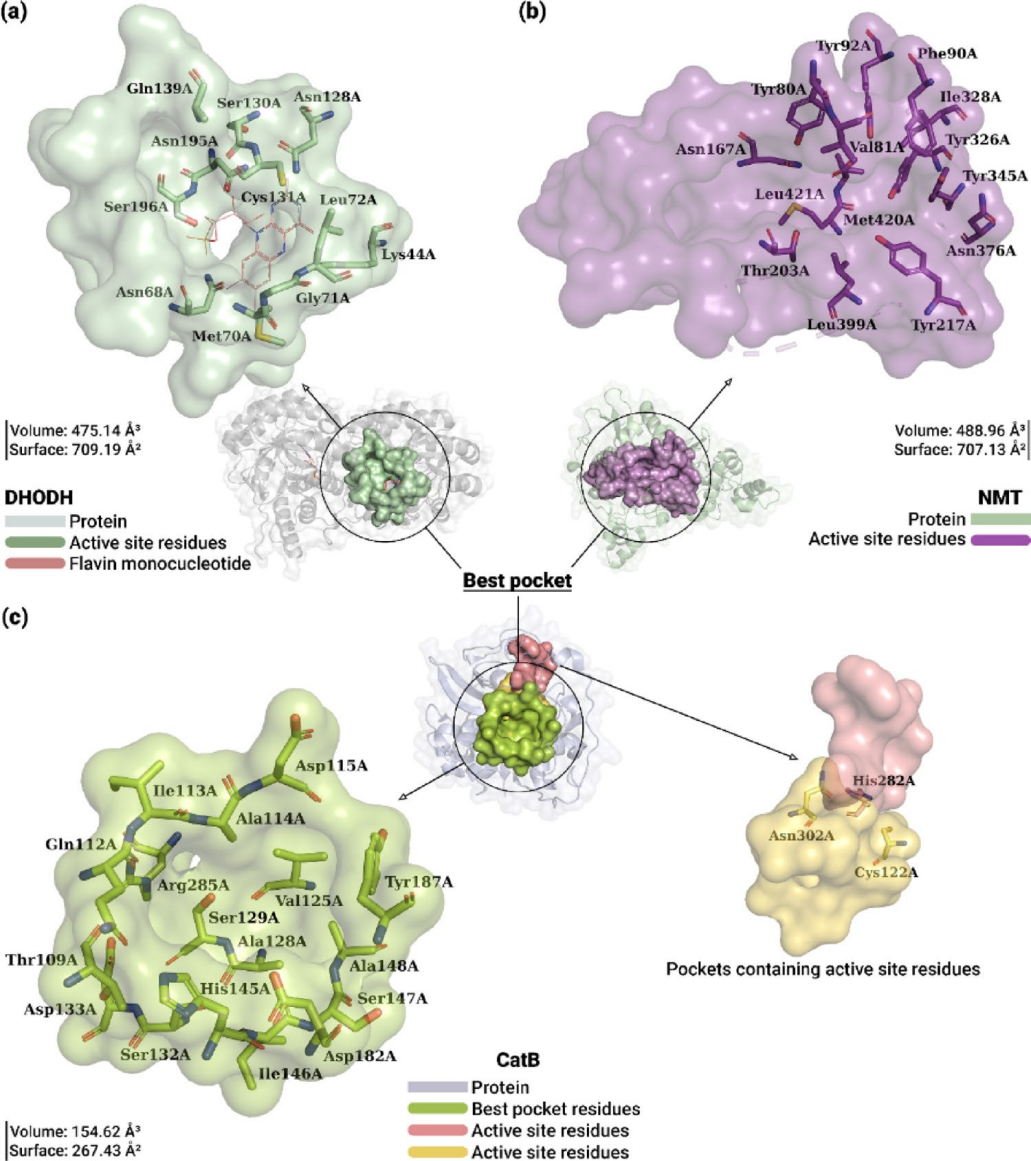
Characterization of the best pocket of the DHODH, NMT, and CatB proteins. **a** − **c** The most significant cavity in each protein is highlighted by a black circle. In **a**, the best pocket of DHODH is shown in light green, highlighting the active site residues and the FMN cofactor (pink). In **b**, the best pocket of NMT is shown in purple, along with the residues that make up the active site. In **c**, the best pocket of CatB is shown in green, along with the residues that compose it. The catalytic site of this protein is distributed across adjacent cavities, highlighted in yellow and pink

The 3D structure of DHODH exhibited a cavity substantially larger than the others, with a volume and surface area estimated at 475.14 Å^3^ and 709.19 Å^2^, respectively. This cavity, shown in Fig. 4a, consists of 43 residues, grouped into aliphatic (20), polar (16), charged (4), and aromatic (3) side chains. In addition, this cavity includes five binding sites, notably the adjacent sites 1 and 2 described by Cheleski et al. (Cheleski et al. 2010). The molecular composition of these sites included residues with polar side chains (Asn68A, Ser100A, Asn107A, Asn128A, Ser130A, Cys131A, Pro132A, Asn133A, Pro138A, Gln139A, Cys150A, Asn195A, and Ser196A), aliphatic (Met70A, Gly71A, Leu72A, Gly101A, Leu102A, Leu129A, Val134A, and Val140A), and positively charged (Lys44A and Lys137A).

Structural characterization of NMT revealed the presence of a prominent cavity located in the functionally relevant region of the protein. This cavity was identified as a potential binding site due to its volume and surface area, calculated at 488.96 Å^3^ and 707.13 Å^2^, respectively, as shown in Fig. 4b. Analysis of the molecular composition revealed that the cavity was formed by 42 residues, distributed among aliphatic (22), polar (8), and aromatic (12) side chains. Within it lies the inhibition site, whose molecular characterization demonstrated a prevalence of aromatic side chains, including Tyr80A, Phe88A, Phe90A, Tyr92A, Tyr217A, Phe232A, Tyr326A, and Tyr345A. In addition, the site included aliphatic residues (Val81A, Leu341A, Ala343A, Val374A, and Leu399A), polar (Asn167A, Thr203A, Ser330A, Asn376A), and charged (Glu82A, His219A, and Asp396A) residues.

CatB, on the other hand, exhibited a cavity predominantly composed of 17 residues with aliphatic side chains (Ile113A, Ala114A, Val125A, Ala128A, Ile146A, and Ala148A), polar (Thr109A, Gln112A, Ser129A, Ser132A, and Ser147A), charged (Asp115A, Asp133A, His145A, Asp182A, and Arg285A), and aromatic (Tyr187) residues, as shown in Fig. 4c. Although this pocket had a volume (154.62 Å^3^) and surface area (267.43 Å^2^) considerably larger than the other cavities, the catalytic trio (Cys122A/His282A/Asn302A) was observed in two different cavities, adjacent to the posterior region of the best pocket.

### Validation of the molecular docking protocol

**Fig. 5:**
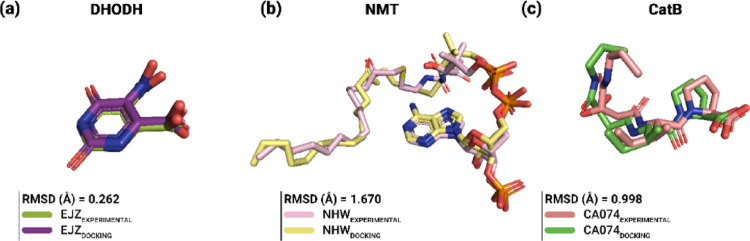
Validation of the molecular docking protocol by redocking of co-crystallized inhibitors. Superposition between the experimental crystallographic poses and the poses reproduced by redocking for **a** DHODH-EJZ, **b** NMT-NHW, and **c** CatB-CA074 complexes. The RMSD values obtained indicate a satisfactory reproduction of the experimental binding modes

Figure 5 shows that the superposition between the experimental crystallographic positions and those reproduced by AutoDock Vina revealed a high degree of structural agreement for all targets evaluated, with RMSD values below 2.0 Å. The lowest RMSD value (experimental vs. simulated conformation) was observed for the DHODH-EJZ complex (0.262 Å), indicating an almost complete reproduction of the crystallographic conformation. The NMT-NHW and CatB-CA074 complexes also showed satisfactory agreement, with RMSD values of 1.670 Å and 0.998 Å, respectively, validating the coupling parameters used in this study.

### Interaction analysis of co-crystallized inhibitors

**Fig. 6:**
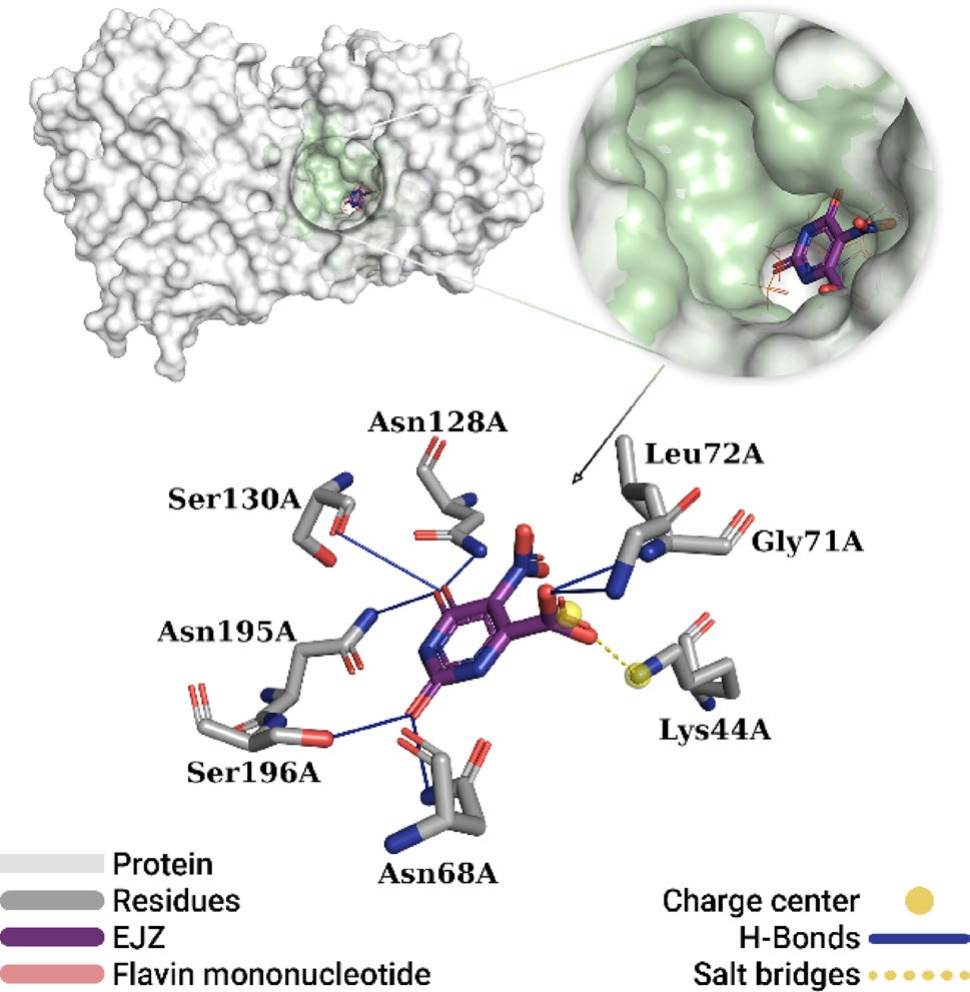
Best pose of the co-crystallized EJZ inhibitor obtained by redocking. The DHODH protein is depicted as a surface (gray), with the best pocket highlighted in light green. Interactions with residues in the pocket include hydrogen bonds (solid blue lines) and salt bridges (dashed yellow line)

Figure 6 shows the global position of the best pose obtained from the redocking of the EJZ inhibitor on the dihydroorotate dehydrogenase protein. The simulation accurately reproduced the experimental pose of the ligand in the best pocket, demonstrating the reliability of the docking protocol. The DHODH-EJZ complex established eight interactions with residues in the active site, consisting of seven hydrogen bonds and one salt bridge interaction. The oxygen (= O) bonded to carbon (− CN_2_H_2_) formed two hydrogen bonds with polar residues: one with the hydrogen (− OH) of the Ser196A side chain (2.00 Å) and one with the hydrogen (− NH_2_) of the side chain of Asp68A (1.89 Å). Additionally, the oxygen (= O) bonded to carbon C4 of the heterocyclic ring formed three hydrogen bonds with hydrogens from the polar side chains of the residues Asp128A (1.80 Å), Ser130A (3.08 Å) and Asp195A (1.81 Å). Two other hydrogen bonds were observed between the oxygen (− COO^-^) of the ligand and the hydrogens (− NH_3_) of residues Gly71A (2.04 Å) and Leu72A (2.07 Å). Finally, a salt-bridge interaction was observed between the carboxyl group of the inhibitor and the positively charged side chain amine of the Lys44A residue (3.88 Å).

**Fig. 7:**
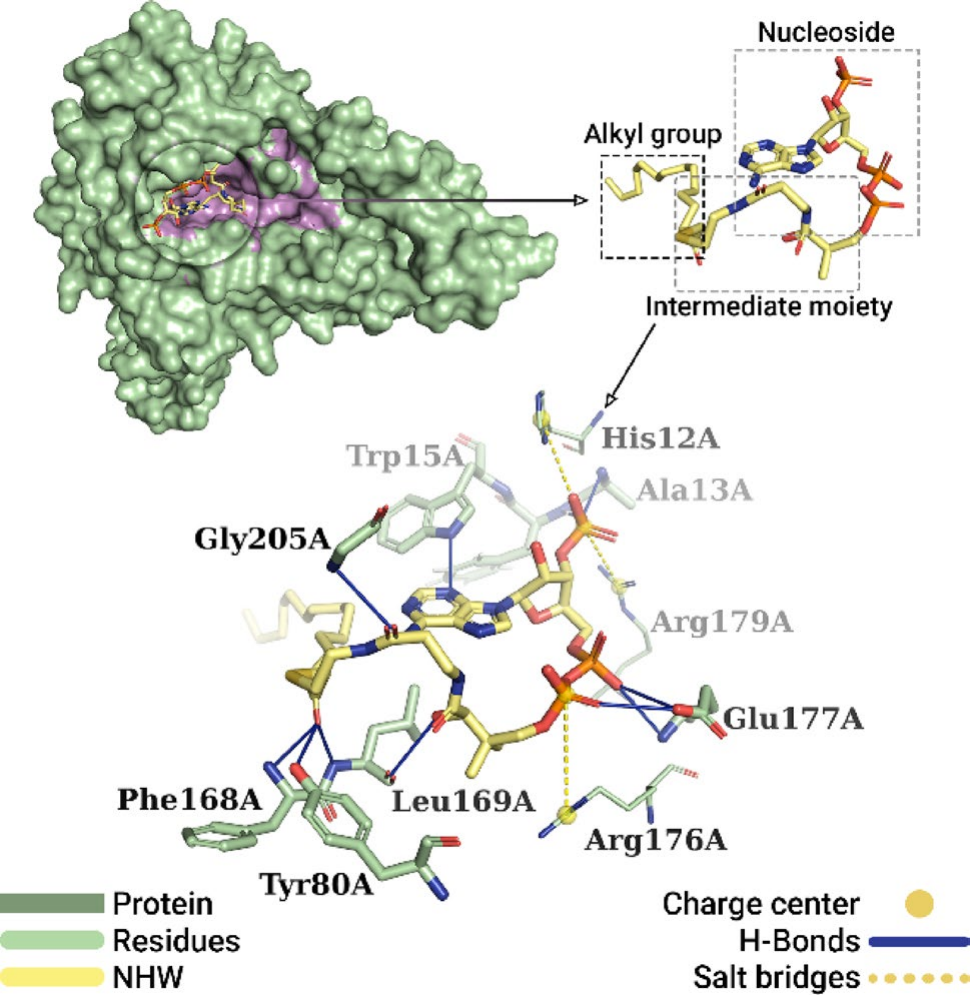
Best pose of the co-crystallized NHW inhibitor obtained by redocking. The NMT is represented as a surface (green), with the best pocket highlighted in purple. Interactions with residues in the pocket include hydrogen bonds (solid blue lines) and salt bridges (dashed yellow line). Hydrophobic interactions have been omitted for better visualization

Concerning the molecular docking simulations conducted with the NHW inhibitor on *N*-myristoyltransferase, the global position corresponding to the best pose obtained can be seen in Fig. 7. The NMT-NHW complex exhibited 31 interactions, notably hydrophobic interactions (17), hydrogen bonds (11), and salt bridge interactions (3). Among the hydrophobic interactions, interactions with aliphatic residues were observed, including Ile166A (3.79 Å, 3.63 Å, and 3.63 Å), Leu169A (3.86 Å), Ile185A (3.93 Å, 3.74 Å, 4.00 Å), Val188A (3.65 Å), Val192A (3.49 Å and 3.79 Å), Ala200A (3.72 Å), and Val206A (3.75 Å). Additionally, hydrophobic interactions with aromatic residues were identified, involving Trp15A (3.65 Å and 3.83 Å), Tyr202A (3.79 Å), and Tyr404A (3.63 Å and 3.64 Å). These interactions occurred primarily with the ligand’s lipid chain.

The hydrogen bonds were distributed across three different regions of the ligand: the nucleoside region, the alkyl chain domain, and the intermediate moiety. In the nucleoside region, an interaction occurred between the hydrogen (− NH) of the adenine molecule and the nitrogen (− NH) of the aromatic side chain of Trp15A (2.26 Å), while two interactions were established between the oxygen (− O^-^) of the phosphate group bound to ribose and the hydrogen (− NH) of residues Ala13A (2.59 Å) and Phe14A (2.08 Å). Also in this region, the oxygen atoms (− OH) and (= O) of the pyrophosphate group interacted with the hydrogen (− OH) of the side chain of the Glu177A residue (2.84 Å and 3.00 Å, respectively). In addition, the oxygen (− OH) also established an interaction with the hydrogen (− NH) of the same Glu177A residue (2.38 Å). In the intermediate moiety of the ligand, two hydrogen bonds were observed: one between the hydrogen (− OH) of the portion and the oxygen of the Leu169A residue (2.30 Å) and another between the carbonyl oxygen (= O) of the ligand and the hydrogen (− NH_3_) of the Gly205A residue (2.52 Å). Finally, in the region corresponding to the alkyl group, the oxygen (= O), bonded to the second carbon of the lipid chain from the sulfur, formed three hydrogen bonds: with the hydrogen (− OH) of the aromatic side chain of the Tyr80A residue (2.24 Å); with the hydrogen (− NH_2_) of the Phe168A residue (2.49 Å); and with the hydrogen (− NH) of the Leu169A residue (2.12 Å).

In terms of salt bridge interactions, all were observed in the nucleoside moiety of the ligand. The phosphate group bound to ribose exhibited two interactions: one with the imidazole moiety of the aromatic side chain of the His12A residue (5.32 Å); and one with the guanidine of the positively charged side chain of the Arg179A residue (4.13 Å). Concurrently, the second phosphate of the pyrophosphate interacted with the imidazole moiety of the aromatic side chain of the His176A residue (5.06 Å).

**Fig. 8:**
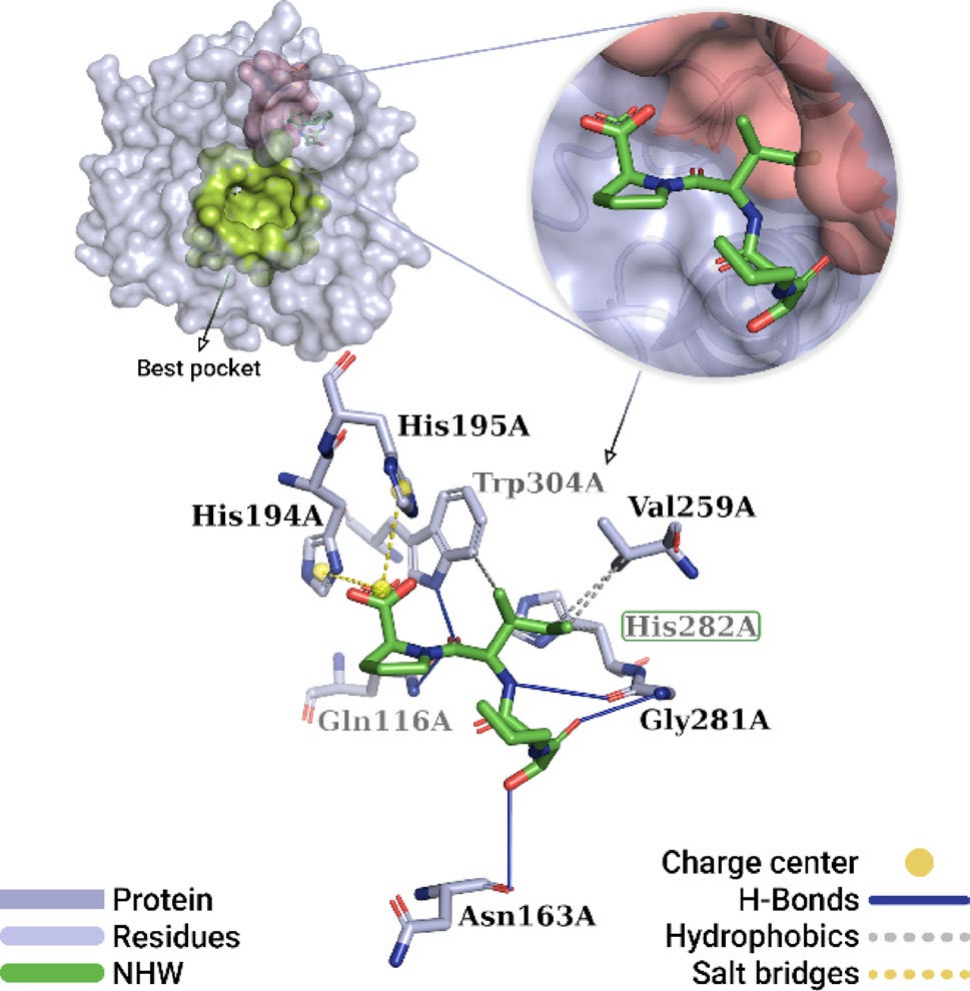
Best pose of the co-crystallized inhibitor CA074 obtained by redocking. The crystallized CatB protein is represented as a surface (lilac), with the best pocket highlighted in green. The adjacent cavity (pink) contains residues from the active site. Interactions with residues in this cavity include hydrophobic interactions (gray dashed lines), hydrogen bonds (solid blue lines), and salt bridges (yellow dashed line)

Regarding the binding between the CA074 inhibitor and cathepsin B, Fig. 8 shows the best pose obtained from the redocking simulations. The ligand reproduced the experimental orientation, binding to the cavity containing the catalytic site of the enzyme. The CatB-CA074 complex exhibited 11 interactions, including four hydrophobic interactions, five hydrogen bonds, and two salt bridge interactions. Among the hydrophobic interactions, the one established between the Cγ (− CH_2_) of the ligand’s isoleucine moiety and the Cβ of the positively charged side chain of the catalytic residue His282A (3.67 Å) stands out. In addition, three other hydrophobic interactions were observed with the side chains of residues Val259A (3.75 Å and 3.79 Å) and Trp304A (3.85 Å).

Related to hydrogen bonds, the proline moiety of the ligand formed two bonds involving oxygen (= O): one with the hydrogen (− NH_2_) of the polar side chain of the Gln116A residue (2.63 Å) and one with the hydrogen (− NH) of the aromatic side chain of the Trp304A residue (2.24 Å). Additionally, three hydrogen bonds were observed involving the butan-dionyl moiety of the ligand: one between the hydrogen (− OH) and the oxygen (− COO^-^) of the Asn163A residue (3.22 Å); one between the oxygen (= O) and the hydrogen (− NH_3_^+^) of the Gly281A residue (2.81 Å); and one between the hydrogen (− NH) of the portion and the oxygen (− COO^-^) of the Gly281A residue (3.15 Å). Finally, the carboxyl group of the proline moiety formed two salt-bridge interactions with the imidazole group of the positively charged side chains of residues His194A (4.39 Å) and His195A (4.23 Å).

### Molecular docking simulations for β-Lapachone-derived compounds

**Fig. 9:**
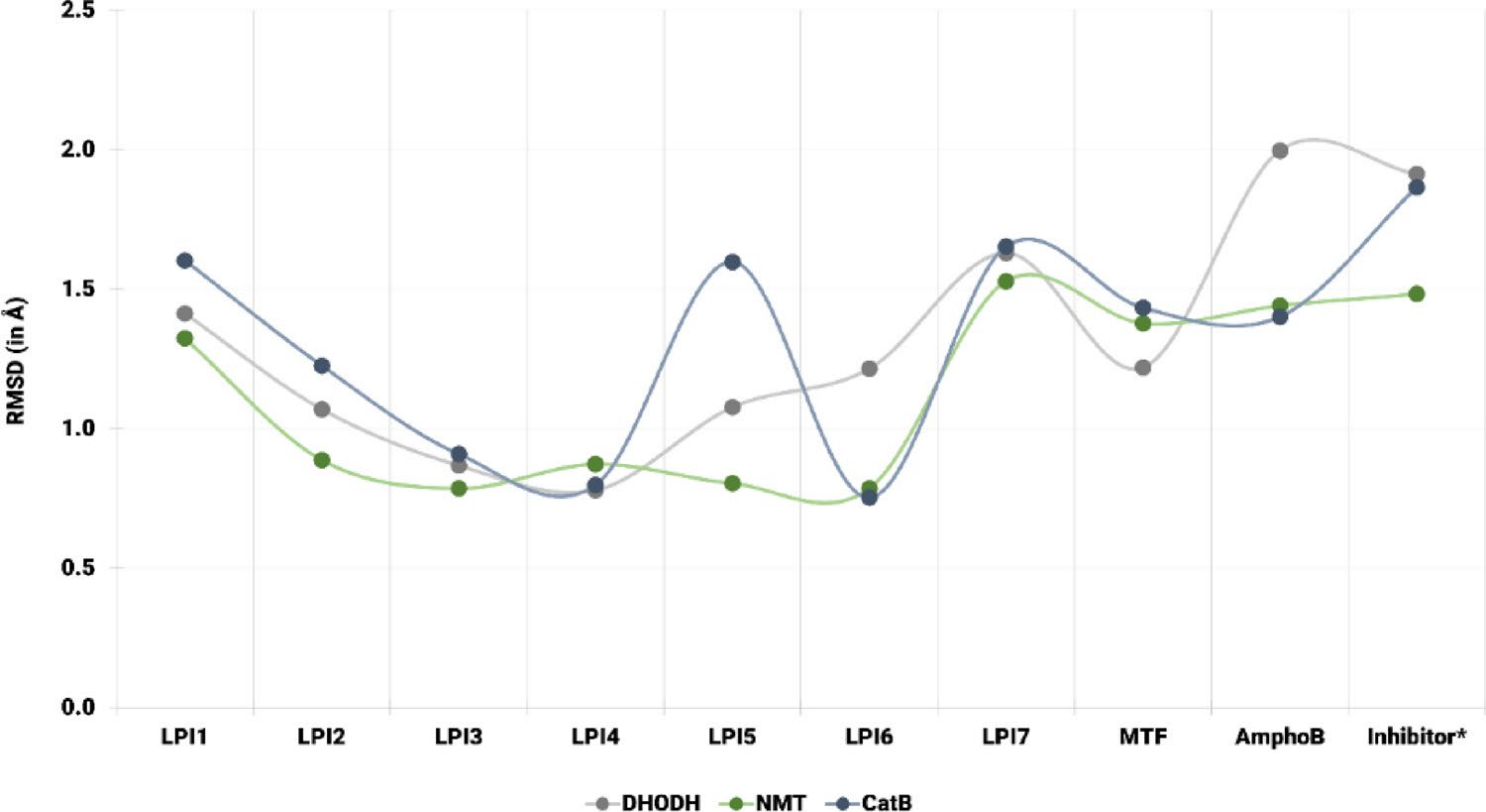
RMSD values (Å) of the protein-ligand complexes for DHODH (gray), NMT (green), and CatB (blue), obtained by molecular docking with the derivatives LPI1–LPI7, the controls MTF and AmphoB, as well as the co-crystallized inhibitors. The symbol * indicates the inhibitors: EJZ for DHODH, NHW for NMT, and CA074 for CatB

 Figure 9 shows the distribution of RMSD (Å) values obtained from molecular docking simulations between ligands LPI1 − LPI7, the reference drugs MTF and AmphoB, as well as the co-crystallized inhibitors, and the 3D structures of the evaluated molecular targets, described in Table S1 (Supplementary Information). In general, the RMSD values remained below 2.0 Å and showed differences dependent on both the ligand and the molecular target. The β-lapachone derivatives exhibited lower values than the control drugs and the inhibitors. Among the derivatives, the lowest values were obtained for compounds LPI3 and LPI4, while the highest were observed for LPI7. Furthermore, when comparing the three molecular targets, it was observed that the complexes formed with DHODH and NMT exhibited the lowest RMSD values and the least variation among ligands, whereas the complexes with CatB demonstrated greater dispersion in the observed values.

Specifically, for the complexes formed with DHODH, the RMSD values ranged from approximately 0.8 Å to 2.0 Å. The highest value observed among all ligands corresponded to the drug AmphoB (1.997 Å), followed by the co-crystallized inhibitor EJZ (1.913 Å). The reference drug MTF had a value of 1.219 Å. Among the derivatives, the lowest values were obtained by the compounds LPI3 (0.868 Å) and LPI4 (0.779 Å). The other compounds had values of 1.413 Å for LPI1, 1.070 Å for LPI2, 1.077 Å for LPI5, 1.215 Å for LPI6, and 1.630 Å for LPI7.

Compared to NMT, the LPI7 ligand exhibited the highest RMSD value among the evaluated ligands, at 1.529 Å. The co-crystallized inhibitor NHW, as well as the reference drugs MTF and AmphoB, exhibited slightly lower values, 1.483 Å, 1.378 Å, and 1.441 Å, respectively. The values obtained for ligands LPI1 − LPI6 ranged from 0.786 Å, observed for compounds LPI3 and LPI6, to 1.324 Å, observed for LPI1. The remaining compounds showed values of 0.888 Å for LPI2, 0.873 Å for LPI4, and 0.804 Å for LPI5.

For CatB, the highest value corresponded to the co-crystallized inhibitor (1.865 Å). The drug MTF showed 1.433 Å, while AmphoB reached 1.401 Å. Among the β-lapachone derivatives, the highest value was observed for compound LPI7 (1.653 Å), while the lowest was obtained for LPI6 (0.753 Å). The remaining compounds exhibited RMSD values ranging from 0.798 Å to 1.603 Å, specifically 1.603 Å for LPI1, 1.226 Å for LPI2, 0.909 Å for LPI3, 0.798 Å for LPI4, and 1.598 Å for LPI5.

**Fig. 10:**
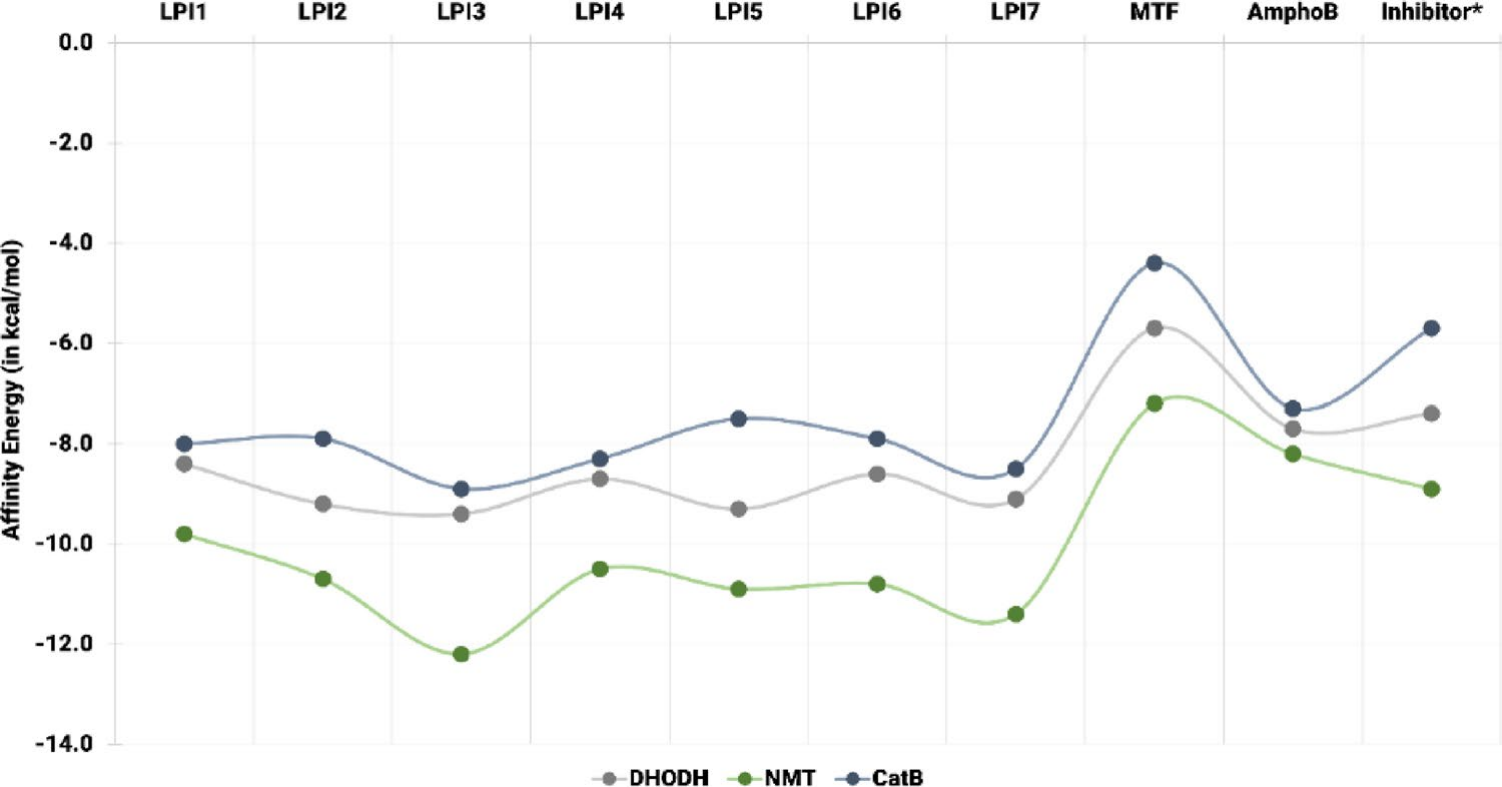
Affinity energy values (kcal/mol) of the protein-ligand complexes for DHODH (gray), NMT (green), and CatB (blue), obtained by molecular docking with the derivatives LPI1–LPI7, the controls MTF and AmphoB, as well as the respective co-crystallized inhibitors. The symbol * indicates the inhibitors: EJZ for DHODH, NHW for NMT, and CA074 for CatB

 Considering E_A_, Fig. 10 presents the results obtained from simulations between the different ligands and molecular targets, as shown in Table S2 (Supplementary Information). In general, the derivatives exhibited considerably lower E_A_ values than those obtained for the controls and co-crystallized inhibitors. The values achieved for the compounds ranged from −7.5 kcal/mol to −12.2 kcal/mol, while those presented by the reference drugs ranged from −4.4 kcal/mol to −8.2 kcal/mol. Although a low dispersion of values was observed among the different derivatives, it was found that the best values across all targets were obtained by the ligand LPI3. In contrast, the highest values corresponded to the drug MTF. Additionally, when comparing the complexes formed by the different molecular targets, it was observed that those formed with NMT had the lowest values, while those formed with CatB had the highest.

In relation to the affinity energies of the DHODH-ligand complexes, the drug MTF exhibited the highest value (−5.7 kcal/mol). The EJZ inhibitor and AmphoB exhibited −7.4 kcal/mol and −7.7 kcal/mol, while the β-lapachone derivatives yielded values ranging from −8.4 kcal/mol to −9.4 kcal/mol. The lowest E_A_ were observed for the ligands LPI3 (−9.4 kcal/mol), LPI5 (−9.3 kcal/mol), LPI2 (−9.2 kcal/mol), and LPI7 (−9.1 kcal/mol).

Regarding the NMT-ligand complexes, the NHW inhibitor had a value of −8.9 kcal/mol, and the control drugs MTF and AmphoB had values of −7.2 kcal/mol and −8.2 kcal/mol, respectively. Among the derivatives evaluated, the values ranged from −9.8 kcal/mol, obtained for LPI1, to −12.2 kcal/mol, for LPI3. The other values observed were −10.7 kcal/mol for LPI2, −10.5 kcal/mol for LPI4, −10.9 kcal/mol for LPI5, −10.8 kcal/mol for LPI6, and −11.4 kcal/mol for LPI7.

As for the CatB-ligand, the E_A_ values of the derivatives were lower than those of the control drugs and the inhibitor CA074. The values obtained for the derivatives ranged from −7.5 kcal/mol to −8.9 kcal/mol, with the best result shown by LPI3, while the highest value corresponded to LPI5. The other compounds had values of −8.0 kcal/mol (LPI1), −7.9 kcal/mol (LPI2), −8.3 kcal/mol (LPI4), −7.9 kcal/mol (LPI6), and −8.5 kcal/mol (LPI7). The drugs MTF and AmphoB, as well as the inhibitor CA074, showed values of −4.4 kcal/mol, −7.3 kcal/mol, and −5.7 kcal/mol, respectively.

**Fig. 11:**
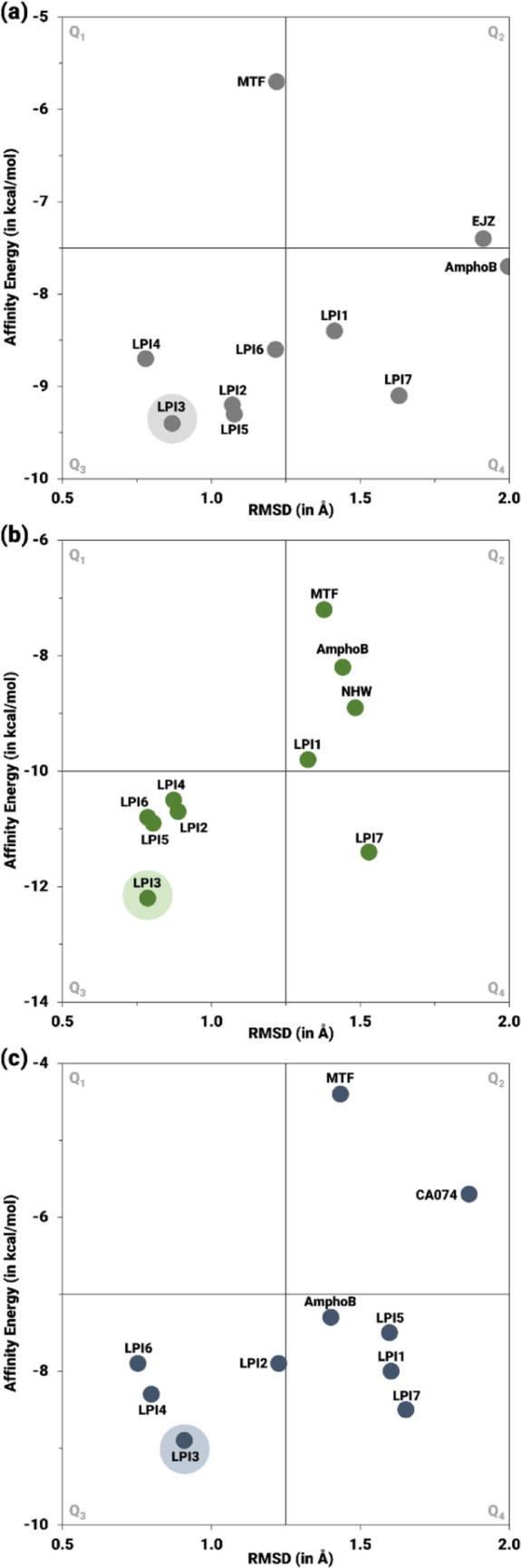
Correlation between RMSD (Å) and EA (kcal/mol) of the protein-ligand complexes for **a** DHODH, **b** NMT, and **c** CatB, obtained by molecular docking with the LPI1–LPI7 derivatives, the MTF and AmphoB controls, and the respective co-crystallized inhibitors (EJZ, NHW, and CA074). Compounds located in Q3 exhibit greater selectivity for the molecular target

The correlation between the RMSD and E_A_ values of the best pose for each compound is shown in Fig. 11. The ligands that exhibited low conformational consistency and low complex stability, indicated by high RMSD and high E_A_, are located in quadrant 2 (Q_2_), while those that exhibited greater selectivity for the DHODH protein, defined by lower RMSD and E_A_, are located in Q_3_. Quadrant Q_1_ indicates compounds with high conformational consistency but low theoretical protein-ligand affinity, while Q_4_ indicates ligands with low conformational consistency and high theoretical affinity. Therefore, with respect to the DHODH protein, Fig. 11a demonstrates that the inhibitor EJZ, due to its higher RMSD and E_A_ values, is distributed in Q_2_. Miltefosine, in turn, can be observed in Q_1_, while the compounds LPI1 and LPI7 and the drug AmphoB are present in Q_4_. The remaining ligands were distributed in Q_3_, notably LPI3, which exhibited low RMSD and the lowest E_A_ among all ligands.

Figure 11b, related to the NMT protein, highlights that the highest values of these parameters were obtained by the NHW inhibitor, MTF, AmphoB, and the LPI1 compound, placing them in Q_2_, while the LPI7 compound is located in Q_4_. The remaining derivatives were distributed in Q_3_, notably LPI3, which exhibited greater structural consistency and higher affinity for *N*-myristoyltransferase compared to the other ligands. As for CatB, shown in Fig. 11c, it is observed that the derivatives LPI2, LPI3, LPI4, and LPI6 were distributed in Q_3_, while the other ligands and AmphoB were located in Q_4_. Similar to the other targets, the ligand LPI3 stood out with the best values. In addition, MTF and the inhibitor CA074 were distributed in Q_2_.

### Interaction analysis between β-lapachone-derived compounds and protein structure

**Fig. 12:**
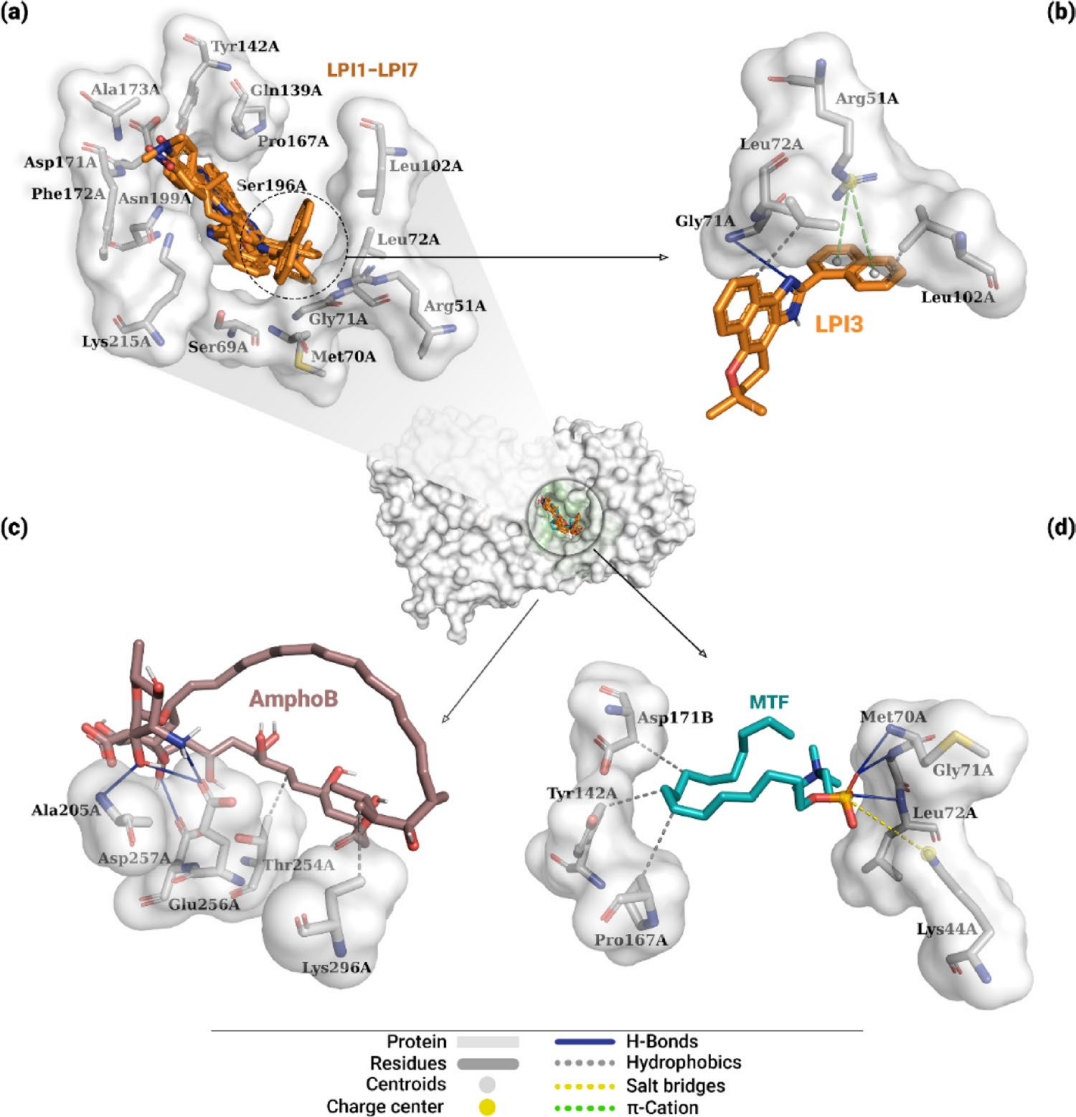
Interactions of DHODH-ligand complexes obtained by molecular docking. The central figure shows the global location of all evaluated ligands. In **a**, the superimposition of the poses of derivatives LPI1–LPI7 (orange) and the residues that interacted. In **b**, interactions of the lead compound LPI3 with protein residues. In **c** and **d**, the interactions of the AmphoB and MTF controls are shown, respectively. Interactions with the residues include hydrophobic interactions (gray dashed lines), hydrogen bonds (solid blue lines), π-cation interactions (green dashed lines), and salt bridges (yellow dashed line)

The main interactions resulting from molecular docking simulations between the different ligands and DHODH can be seen in Fig. 12. Based on the global position of the ligands on the protein, indicated by the 3D structure in the center of this figure, it was observed that compounds LPI1–LPI7 and the drug MTF bound to the protein’s best pocket, while AmphoB was located in a different pocket. The evaluated derivatives interacted with 14 protein residues, as shown in Fig. 12a, particularly the aliphatic residues Met70A, Gly71A, Leu72A, and Leu102A, and the polar residues Gln139A and Ser196A, which are part of the active site of dihydroorotate dehydrogenase. Table 1 describes the types of interactions between the lead compound, as well as the reference drugs, and DHODH.

Based on the analysis of ligand-DHODH interactions for the lead compound LPI3, five interactions were identified in the complex (Table 1). A hydrophobic interaction was observed between the aromatic carbon of the naphthalene moiety and the Cγ of the aliphatic side chain of the Leu72A residue (3.83 Å), shown in Fig. 12b. The second hydrophobic interaction occurred between the aromatic carbon of the naphthalene substituent and the methyl carbon of the aliphatic side chain of the Leu102A residue (3.41 Å). In addition, the pyridinium nitrogen of the imidazole moiety formed a hydrogen bond with the hydrogen (− NH_3_^+^) of the Gly71A residue (3.57 Å). Regarding the substituent, two π-cation interactions were observed between the aromatic rings of the moiety and the guanidine group of the positively charged side chain of residue Arg51A (4.90 Å and 4.65 Å).

**Table 1:** Interaction types between ligands and Dihydroorotate dehydrogenase

Ligand	Interaction type	Residue (Distance in Å)
LPI3	Hydrophobic	Leu72A (3.83), Leu102A (3.41)
	H-Bond	Gly71A (3.57)
	π-Cation	Arg51A (4.90), Arg51A (4.65)
MTF*	Hydrophobic	Tyr142A (3.45), Pro167A (3.89), Asp171B (3.77)
	H-Bond	Met70A (2.27), Gly71A (2.23), Leu72A (2.48)
	Salt Bridges	Lys44A (5.26)
AmphoB*	Hydrophobic	Thr254A (3.29), Lys296A (3.67)
	H-Bond	Ala205B (2.03), Glu256A (2.35), Glu256A (2.48), Asp257A (2.13)

*Control

 For the other compounds, Table S3 (Supplementary Information) shows the main interactions established with the DHODH protein. Overall, the naphthalene moiety of these derivatives exhibited hydrophobic interactions with residues Gln139A, Tyr142A, Pro167A, Asp171B, and Lys215A. The dihydropyran ring, in turn, exhibited hydrophobic interactions with residues Ser69A and Met70A, as well as hydrogen bonds with residues Asn199A and Lys215A. As for the imidazole moiety, hydrogen bonds were observed with the side chains of the polar residues Ser196A and Asn199A. And, despite the different structures corresponding to the substituents, these moieties exhibited hydrophobic interactions with only five residues: Met70A, Gln139A, Asp171B, Phe172B, and Ala173B.

In the case of the control ligand AmphoB, shown in Fig. 12c, six interactions with the target protein were observed, including two hydrophobic interactions between main chain carbons and the methyl carbon of the polar side chain of the Thr254A residue (3.29 Å) and with the Cγ of the positively charged side chain of the Lys296A residue (3.67 Å), as well as four hydrogen bonds (Table 1). These interactions occurred primarily with residues possessing positively charged side chains: Glu256A and Asp257A. Only one interaction was established with residues containing aliphatic side chains, namely Ala205B. Two interactions were established involving the hydroxyl group of mycosamine: one with the oxygen (− COO^-^) of the negatively charged side chain of residue Glu256A (2.48 Å) and one with the hydrogen (− NH) of residue Ala205B (2.03 Å). A hydrogen bond was also observed between the hydrogen (− NH_3_) and the oxygen (− COO^-^) of the side chain of the Glu256A residue (2.35 Å). Finally, an interaction was established between the hydrogen (− OH) attached to the main chain and the oxygen (− COO^-^) of the negatively charged side chain of the Asp257A residue (2.13 Å).

The reference drug MTF, shown in Fig. 12d, exhibited seven interactions with the DHODH protein, including three hydrophobic interactions, three hydrogen bonds, and one salt bridge interaction (Table 1). The alkyl group of the ligand established hydrophobic interactions with an aromatic carbon in the side chain of the Tyr142A residue (3.45 Å), with a carbon (− CH_2_) atom from the aliphatic side chain of the Pro167A residue (3.89 Å), and with the Cβ atom of the negatively charged side chain of the Asp171B residue (3.77 Å). Hydrogen bonds formed between the oxygen atoms of the phosphate group and the (− NH_3_^+^) hydrogen atoms of residues Met70A (2.27 Å), Gly71A (2.23 Å), and Leu72A (2.48 Å). Additionally, a salt bridge interaction was established between the phosphate group and the amino group of the side chain of the Lys44A residue (5.26 Å).

**Fig. 13:**
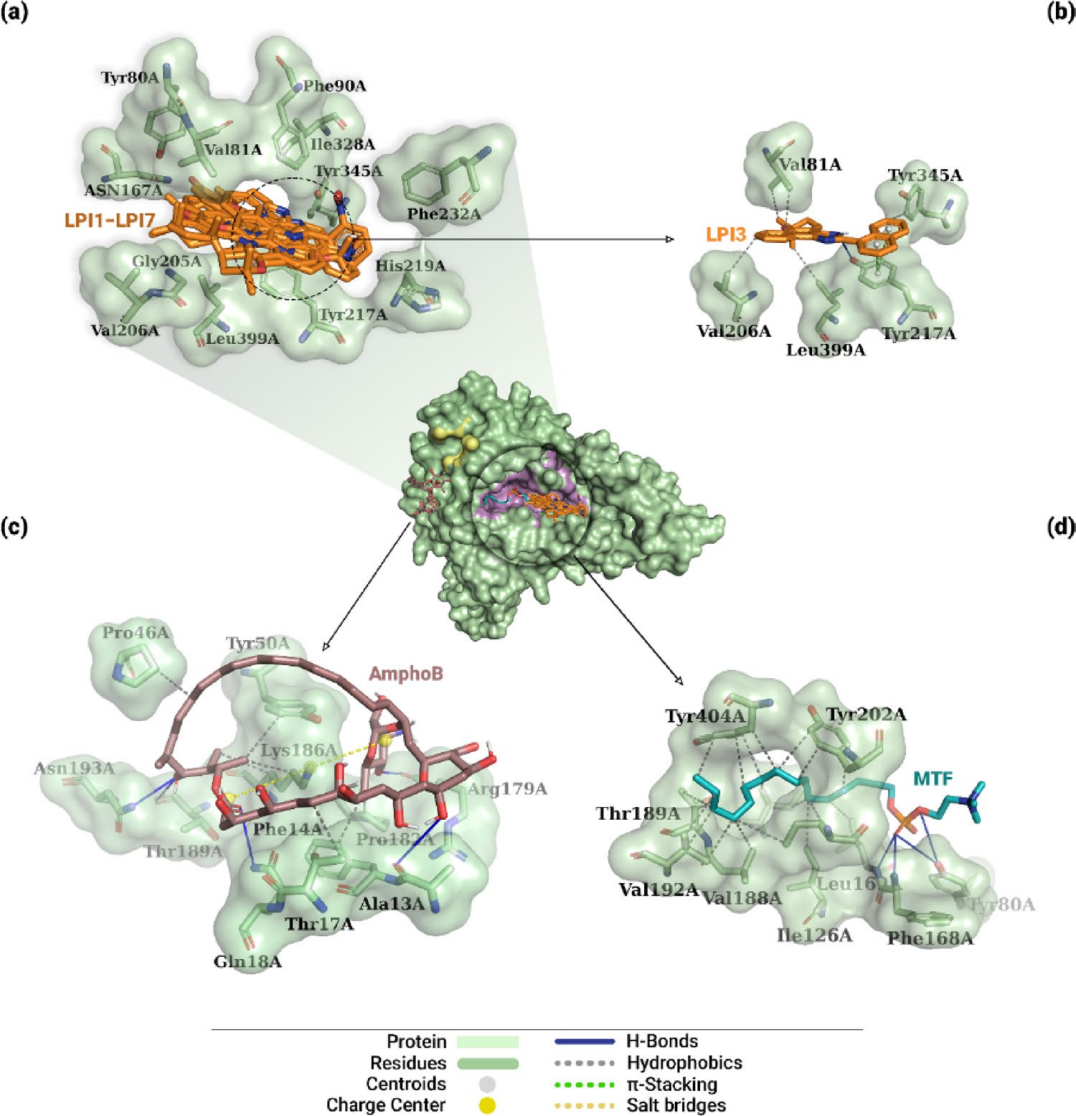
Interactions of NMT-ligand complexes obtained by molecular docking. The central figure shows the global location of all evaluated ligands. In **a**, the superimposition of the poses of the LPI1–LPI7 derivatives (orange) and the residues that interacted. In **b**, interactions of the lead compound LPI3 with protein residues. In **c** and **d**, the interactions of the AmphoB and MTF controls are shown, respectively. Interactions with the residues include hydrophobic interactions (gray dashed lines), hydrogen bonds (solid blue lines), π-stacking (green dashed lines), and salt bridges (yellow dashed line)

Regarding the NMT-ligand complexes, Fig. 13 shows the best pose for each ligand, the main interactions established, and the respective residues involved in these interactions. The global structure of the protein, including the distribution of the best poses of the ligands, demonstrates that compounds LPI1–LPI7 and MTF interacted with the best pocket of *N*-myristoyltransferase, while AmphoB interacted with residues adjacent to the same pocket. Furthermore, as shown in Fig. 13a, it is observed that compounds LPI1–LPI7 interacted with 12 residues, of which Tyr80A, Val81A, Phe90A, Asn167A, Tyr217A, His219A, Phe232A, Tyr345A, and Leu399A comprise the protein’s active site. The types of these interactions between the lead compound, as well as the drugs MTF and AmphoB, and the NMT protein are described in Table 2.

Analysis of the interactions revealed that the LPI3 ligand formed five hydrophobic interactions, one hydrogen bond, and two π-stacking interactions with the protein, as shown in Fig. 13b. Among the hydrophobic interactions, two were established between the naphthalene moiety and the terminal methyl carbons of the aliphatic side chains of residues Val81A (3.56 Å) and Val206A (3.76 Å). In addition, the methyl carbons bound to the dihydropyran ring interacted with the terminal methyl carbon of the aliphatic side chain of residues Val81A (3.96 Å) and Leu399 (3.80 Å). Furthermore, a hydrophobic interaction was observed between the aromatic carbon of the substituent and the aromatic carbon of the Tyr345A residue (3.62 Å). Regarding hydrogen bonding, such an interaction was observed between the hydrogen (− NH) and the oxygen (− OH) of the phenolic portion of the aromatic side chain of the Tyr217A residue (3.00 Å). Furthermore, the naphthalene substituent exhibited two parallel π-stacking interactions with the aromatic side chain of the Tyr217A residue (3.91 Å and 4.19 Å) (Table 2).

**Table 2:** Interaction types between ligands and *N*-myristoyltransferase

Ligand	Interaction type	Residue (Distance in Å)
LPI3	Hydrophobic	Val81A (3.96), Val81A (3.56), Val206A (3.76), Tyr345A (3.62), Leu399A (3.80)
	H-Bond	Tyr217A (3.00)
	π-Stacking	Tyr217A (3.91), Tyr217A (4.19)
MTF*	Hydrophobic	Ile126A (3.68), Ile166A (3.88), Ile166A (3.67), Leu169A (3.83), Ile185A (3.50), Ile185A (3.47), Val188A (3.79), Thr189A (3.87), Val192A (3.78), Val192A (3.73), Val192A (3.47), Tyr202A (3.35), Tyr202A (3.66), Tyr202A (3.74), Tyr404A (3.66), Tyr404A (3.85), Tyr404A (3.63), Tyr404A (3.55)
	H-Bond	Tyr80A (2.74), Tyr80A (3.05), Phe168A (2.82), Leu169A (2.14)
AmphoB*	Hydrophobic	Phe14A (3.75), Thr17A (3.68), Pro46A (3.55), Tyr50A (3.94), Pro182A (3.64), Lys186A (3.84), Lys186A (3.5)
	H-Bond	Ala13A (3.2), Gln18A (2.78), Arg179A (2.23), Thr189A (3.59), Asn193A (2.84)
	Salt Bridges	Lys186A (5.17 Å), Lys186A (4.64 Å)

*Control

 The main interactions between the other compounds and NMT are described in Table S4 (Supplementary Information). In summary, the naphthalene moiety of these derivatives exhibited hydrophobic interactions with residues Val81A, Phe90A, and Tyr217A, as well as parallel π-stacking interactions with residue Tyr217A. The dihydropyran ring exhibited hydrophobic interactions with residues Gly205A, Val206A, Ile328A, and Tyr345A. As for the imidazole moiety, the ligands formed a hydrogen bond with residue Tyr345A and a parallel π-stacking interaction with residue Tyr217A. As for the substituents, hydrophobic interactions were observed with residues Tyr80A, Val81A, Tyr217A, His219A, and Phe232A, hydrogen bonds with residue Asn167A, and parallel π-stacking with residue Tyr217A.

In the case of the reference drug AmphoB, shown in Fig. 13c, its main chain formed seven hydrophobic interactions: one with the carbon (− CH_2_) of the aliphatic chain of the Pro46A residue (3.55 Å); two with the Cβ and Cδ of the positively charged side chain of residue Lys186A (3.84 Å and 3.50 Å, respectively); one with the aromatic carbon of the side chain of residue Tyr50A (3.94 Å); one with the Cβ of the aliphatic side chain of residue Pro182A (3.64 Å); one with the aromatic carbon of the side chain of residue Phe14A (3.75 Å); and one with the methyl carbon of the polar side chain of residue Thr17A (3.68 Å). Additionally, five hydrogen bonds were identified: one between the hydrogen (− OH) bound to the main chain and the oxygen of the Ala13A residue (3.20 Å); one between the hydrogen (− OH) of mycosamine and the oxygen of the Arg179A residue (2.23 Å); one between the oxygen (= O) attached to the main chain and the hydrogen (− NH_2_) of the polar side chain of the Gln18A residue (2.78 Å); and two from the hydroxyl group bound to the main chain, one with the oxygen of the Thr189A residue (3.59 Å) and one with the hydrogen (− NH_2_) of the polar side chain of the Asn193A residue (2.84 Å). Additionally, two salt bridge interactions were observed between carboxyl groups and the NH_3_^+^ group of the positively charged side chain of the Lys186A residue (5.17 Å and 4.64 Å).

Figure 13d shows that the reference drug MTF had 22 interactions with NMT. The alkyl group of the ligand had 18 hydrophobic interactions with the protein: ten interactions with the aliphatic side chains of residues Ile126A (3.68 Å), Ile166A (3.88 Å and 3.67 Å), Leu169A (3.83 Å), Ile185 (3.50 Å and 3.47 Å), Val188A (3.79 Å), and Val192A (3.78 Å, 3.73 Å, and 3.47 Å); one interaction with the polar side chain of the Thr189A residue (3.87 Å); and seven interactions with the aromatic side chains of residues Tyr202A (3.35 Å, 3.66 Å, and 3.74 Å) and Tyr404A (3.66 Å, 3.85 Å, 3.63 Å, and 3.55 Å). Furthermore, four hydrogen bonds were formed from the oxygen atoms of the phosphate group, of which two were with the hydroxyl group (− OH) of the aromatic side chain of residue Tyr80A (2.74 Å and 3.05 Å), one with the hydrogen (− NH) of the Phe168A residue (2.82 Å), and one with the hydrogen (− NH) of the Leu169A residue (2.14 Å).

**Fig. 14:**
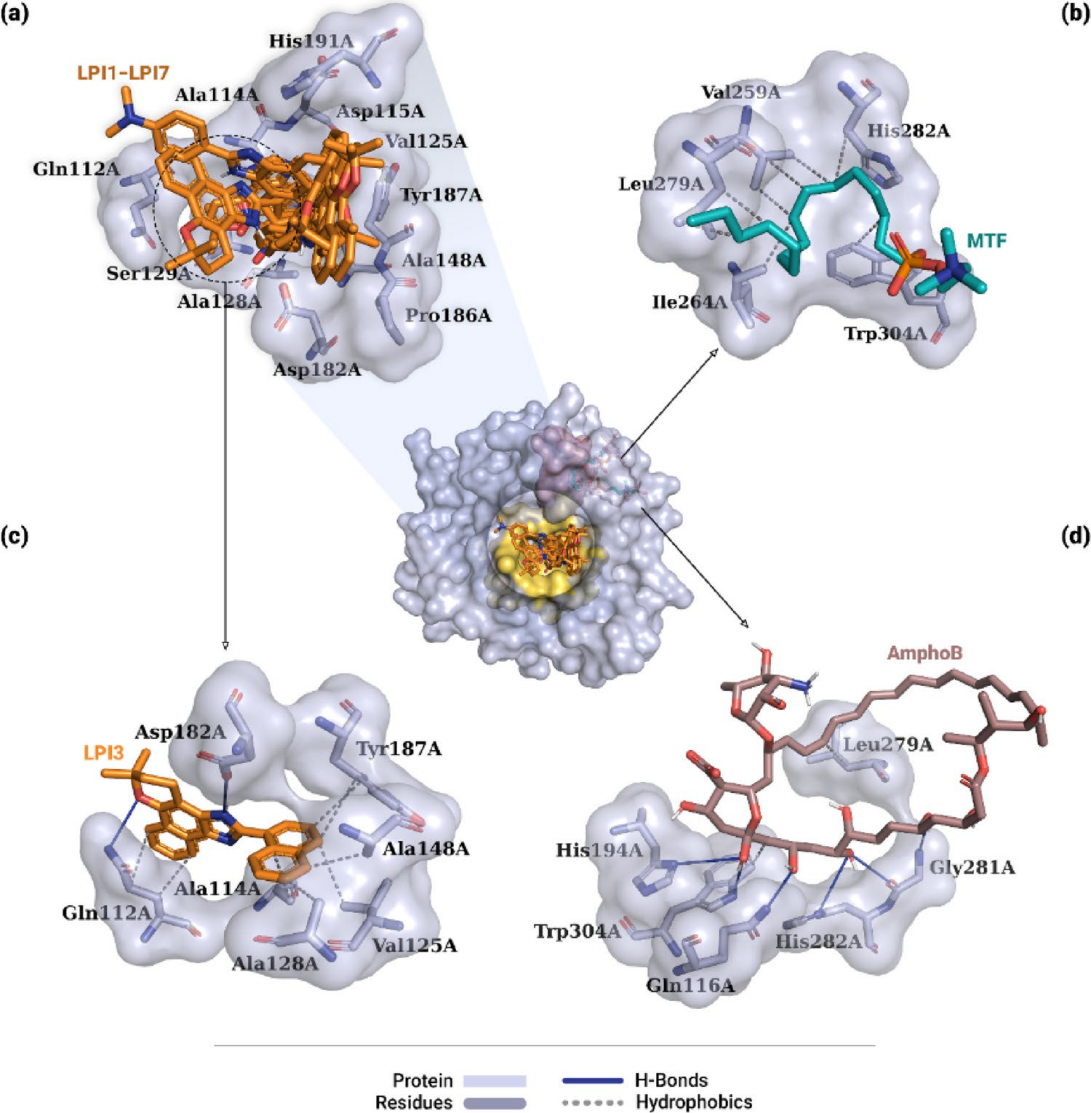
Interactions of CatB-ligand complexes obtained by molecular docking. The central figure shows the global location of all evaluated ligands. In **a**, the superimposition of the poses of derivatives LPI1–LPI7 (orange) and the residues with which they interacted. In **c**, interactions of the lead compound LPI3 with protein residues. In **b** and **d**, the interactions of the AmphoB and MTF controls, respectively, with the adjacent pocket are shown. Interactions with the residues include hydrophobic interactions (gray dashed lines) and hydrogen bonds (solid blue lines)

Figure 14 illustrates the global best pose of the ligands and the residues involved in the interactions identified from molecular docking simulations with the CatB protein. Although compounds LPI1–LPI7 bound to the protein’s best pocket, only the MTF and AmphoB controls interacted with residues in the catalytic site. As shown in Fig. 11a, the evaluated derivatives interacted with 11 residues, distributed among aliphatic (Ala114A, Val125A, Ala128A, Ala148A, and Pro186A), polar (Gln112A and Ser129A), aromatic (Tyr187A), and charged (Asp115A, Asp182A, and His191A). The types of these interactions for the lead compound and the controls are described in Table 3.

The analysis of the interactions between the LPI3 ligand and CatB is shown in Fig. 14c, indicating ten interactions, of which eight were hydrophobic interactions and two were hydrogen bonds. In terms of hydrophobic interactions, two were observed between aromatic carbons of the naphthalene moiety and the Cβ and Cγ carbons of the polar side chain of the Gln112A residue (3.83 Å and 3.75 Å, respectively). Additionally, six interactions were observed involving aromatic carbons of the naphthalene substituent with methyl carbons of the aliphatic side chains of residues Ala114A (3.96 Å), Val125A (3.63 Å), Ala128A (3.48 Å), and Ala148A (3.49 Å), and with aromatic carbons of the side chain of the Tyr187A residue (3.78 Å and 3.93 Å). Regarding hydrogen bonds, one was observed between the oxygen of the dihydropyran ring and the hydrogen (− NH_2_) of the polar side chain of the Gln112A residue (2.93 Å) and one between the hydrogen (− NH) of the imidazole moiety and the oxygen (− COO^-^) of the negatively charged side chain of the Asp182A residue (2.96 Å) (Table 3).

 Table S5 (Supplementary Information) describes the main interactions between the other β-lapachone derivatives and CatB. In general, the naphthalene moiety of these compounds exhibited hydrophobic interactions with residues Ala114A, Asp115A, Val125A, Ala148A, Pro186A, and Tyr187A, and π-cationic interactions with residue His191A. The dihydropyran ring exhibited hydrophobic interactions with residues Gln112A, Val125A, and Pro186A, and a hydrogen bond with residue Ser129A. The imidazole moiety, meanwhile, formed hydrogen bonds with residues Asp115A, Asp182A, and Pro186A. Finally, the substituents exhibited hydrophobic interactions with residues Gln112A, Val125A, Ala128A, Ala148A, and Tyr187A, and hydrogen bonds with residues Val125A and Ser129A.

About the CatB-MTF complex, eight hydrophobic interactions involving carbons from the ligand’s alkyl group were observed (Table 3). Figure 14b shows that three interactions were established with the Cβ and with the terminal methyl carbons of the aliphatic side chain of residue Val259A (3.90 Å, 3.46 Å, and 3.84 Å, respectively). Moreover, one interaction was observed with the Cδ of the aliphatic side chain of the Ile264A residue (3.70 Å), two interactions with the terminal methyl carbons of the aliphatic side chain of the Leu279A residue (3.63 Å and 3.52 Å), one with the Cβ of the positively charged side chain of the His282A residue (3.93 Å), and one with the aromatic carbon of the side chain of the Trp304A residue (3.71 Å).

For the drug AmphoB, Table 3 describes the interactions established with the target protein. The CatB-AmphoB complex formed nine interactions, of which three were hydrophobic interactions and six were hydrogen bonds, as shown in Fig. 14d. The hydrophobic interactions were observed involving carbons from the ligand’s main chain. Two interactions were established with the Cβ and the methyl carbon of the aliphatic side chain of the Leu279A residue (3.53 Å and 3.49 Å). The third interaction was formed with the aromatic carbon of the Trp304A residue (3.32 Å). Regarding hydrogen bonds, two interactions were observed involving hydroxyl groups linked to the main chain, one with the oxygen and the other with the hydrogen (− NH) of the Gly281A residue (3.09 Å and 2.02 Å, respectively). The oxygen (− OH) interacted with the hydrogen (− NH) of the positively charged side chain of the His282A residue (3.35 Å). An interaction was also observed between the oxygen (= O) bonded to the main chain and the hydrogen (− NH_2_) of the polar side chain of the Gln116A residue (2.28 Å). Finally, two interactions were observed with the hydrogen (− NH) of the side chains of residues His194A (3.10 Å) and Trp304A (3.17 Å).

**Table 3:** Interaction types between ligands and Cathepsin B

Ligand	Interaction type	Residue (Distance in Å)
LPI3	Hydrophobic	Gln112A (3.83), Gln112A (3.75), Ala114A (3.96), Val125A (3.63), Ala128A (3.48), Ala148A (3.49), Tyr187A (3.78), Tyr187A (3.93)
	H-Bond	Gln112A (2.93), Asp182A (2.96)
MTF*	Hydrophobic	Val259A (3.9), Val259A (3.46), Val259A (3.84), Ile264A (3.7), Leu279A (3.63), Leu279A (3.52), His282A (3.93), Trp304A (3.71)
AmphoB*	Hydrophobic	Leu279A (3.53), Leu279A (3.49), Trp304A (3.32)
	H-Bond	Gln116A (2.28), His194A (3.1), Gly281A (3.09), Gly281A (2.02), His282A (3.35), Trp304A (3.17)

*Control

### NMA-based conformational dynamics analysis

**Fig. 15:**
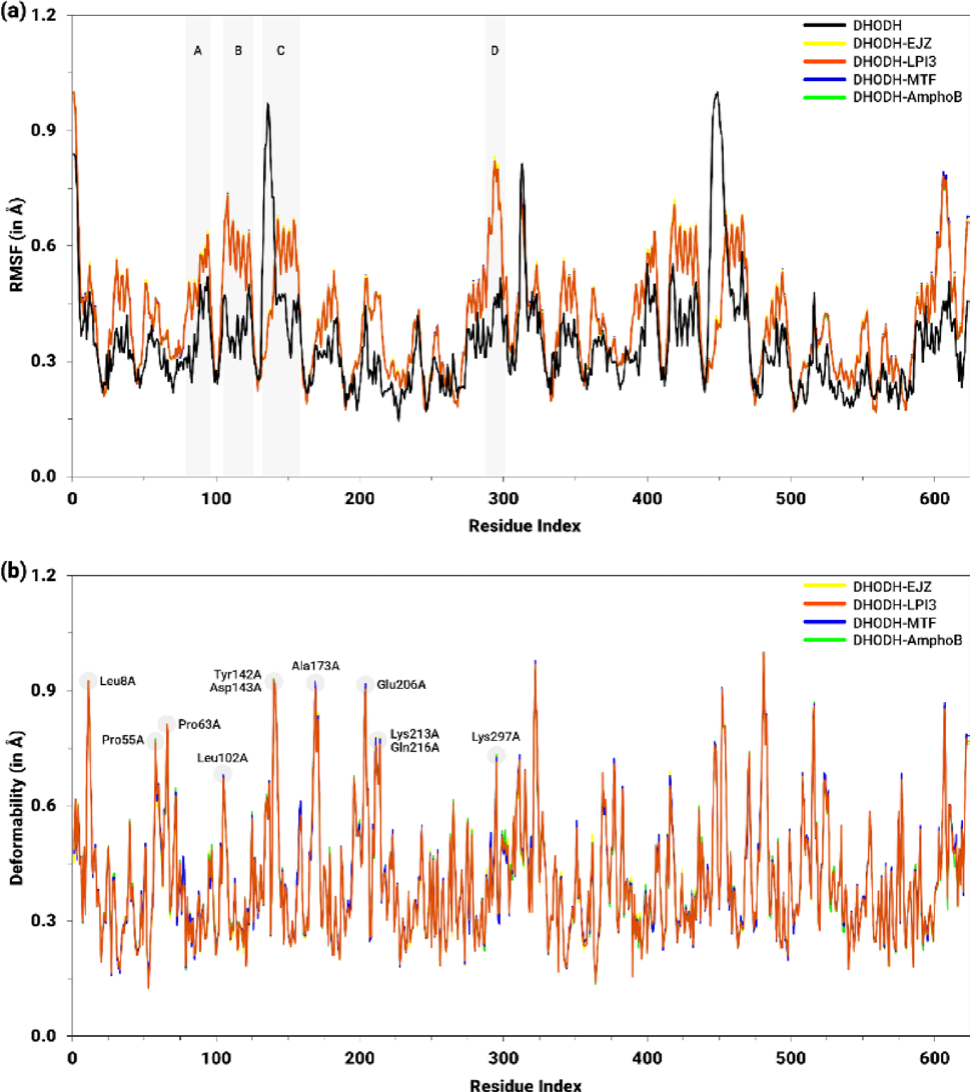
Flexibility (RMSF, in Å) and deformability (Å) profiles per residue for DHODH in its isolated form and in complexes with the inhibitor EJZ (yellow), the derivative LPI3 (orange), and the drugs MTF (blue) and AmphoB (green). In **a**, the shaded regions (A–D) highlight segments with significant differences in flexibility. In **b**, the highlighted peaks indicate the residues with the highest deformability

The flexibility and deformability profiles of the protein-ligand complexes between the compound LPI3, the reference drugs MTF and AmphoB, as well as the co-crystallized inhibitor EJZ, and the target DHODH, obtained from NMA-based conformational dynamics analysis, are shown in Fig. 15. The results for flexibility demonstrated that the NMA module applied to the crystallized DHODH structure, indicated by the black line, exhibited an RMSF ranging from 0.15 Å and 1.0 Å, with the greatest conformational variation in Cα observed for the Leu128A−Asp143A amino acid sequence (C, 0.97 Å), composed mainly of active site residues (Fig. 15a). In general, the other regions of the protein exhibited small variations along the chain, with values ranging from 0.2 Å to 0.5 Å.

 In contrast, it was observed that applying the NMA field in the presence of ligands revealed greater displacement of residues along the entire chain, with the exception of the Leu128A−Asp143A sequence, whose average value (0.35 Å) was substantially lower than that observed for the isolated protein. Consistently, other regions associated with the active site, detailed in Table S6 (Supplementary Information), corresponding to the amino acid sequences Val42A−Lys44A, Gly65A−Pro73A, Phe96A−Glu109A, and Ile191A−Ser196A, exhibited local minima of flexibility, with values similar to those of DHODH in the absence of ligands. However, the greatest structural variations in the complexes were observed in regions A (Gly76A−Tyr83A), B (Val108A−Lys121A), C (Tyr142A−Pro159A), and D (Ser288A−Thr301A), being slightly more pronounced in the complexes with MTF and with the co-crystallized inhibitor EJZ, as shown in Fig. 15a.

Concerning deformability, Fig. 15b shows that all protein-ligand complexes exhibited highly similar profiles across the entire length of the protein. Generally, deformability values ranged from 0.2 Å to 0.6 Å, with isolated peaks near 0.9 Å. These larger displacements were observed mainly in sequences that also exhibited high or moderate conformational variations in RMSF values, especially at residues Leu8A (0.92 Å), Pro55A (0.75 Å), Pro63 (0.80 Å), Leu102A (0.67 Å), Tyr142A (0.92 Å), Asp143A (0.91 Å), Ala173A (0.84 Å), Glu206A (0.91 Å), Lys213A (0.76 Å), Gln216A (0.77 Å), and Lys297A (0.73 Å). Furthermore, although the profiles are generally similar, the values exhibited by the LPI3 compound were slightly lower compared to the control drugs. Furthermore, it was found that the patterns of flexibility and deformability observed in the A chain of the homodimer were reproduced in the equivalent residues of the B chain, regardless of the presence of ligands or the region of interaction with the protein.

**Fig. 16:**
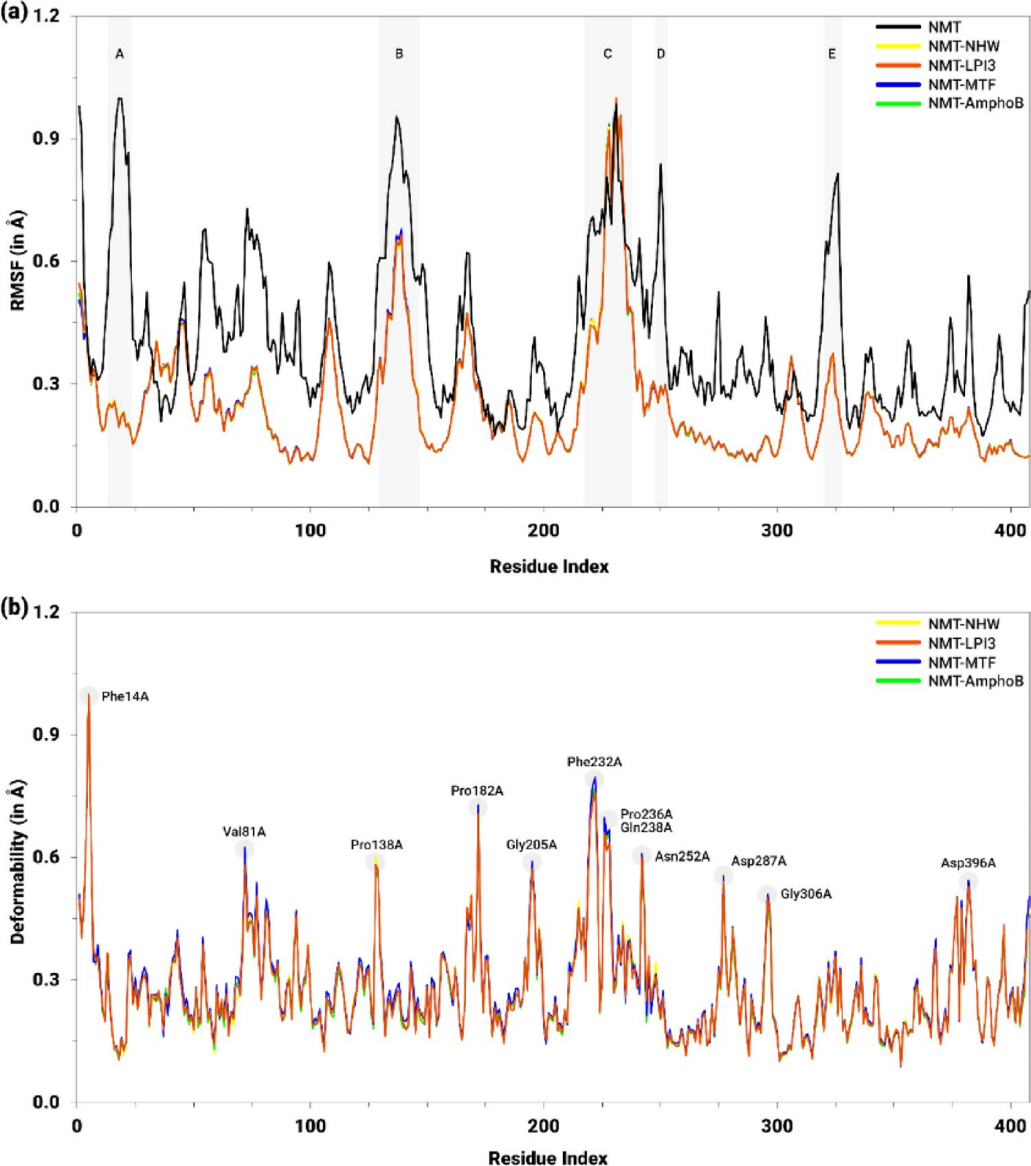
Profile of flexibility (RMSF, in Å) and deformability (Å) per residue for NMT in its isolated form and in complexes with the inhibitor NHW (yellow), the derivative LPI3 (orange), and the drugs MTF (blue) and AmphoB (green). In **a**, the shaded regions (**A**–**E**) highlight segments with significant differences in flexibility. In **b**, the highlighted peaks indicate the residues with the highest deformability

As for the NMT-ligand complexes, the RMSF values of the isolated protein ranged mainly between 0.2 Å and 0.5 Å (Fig. 16a). However, the greatest conformational variations in Cα were observed in the segments corresponding to the residues Gln22A−Phe32A (A), Tyr140A−Glu153A (B), Glu219A−Ala247A (C), Pro259A−Asn261A (D), and Thr331A−Ile340A (E), ranging from 0.8 Å to 1.0 Å, while the sequences Asn62A−Asp68A, Tyr80A−Tyr92A, Lys117A−Lys120A, and Arg176A−Lys178A exhibited intermediate fluctuations, with values between 0.6 Å and 0.7 Å.

 Considering the NMA module applied to the NMT-ligand complexes, with the exception of the Ala237A−Asn244A segment, greater stability was observed throughout the entire protein, characterized by a lower RMSF, compared to isolated NMT (Fig. 16a). The values remained predominantly between 0.15 Å and 0.30 Å, with the Glu147A−Gly149A and Gln240A−Gln243A regions being the most prominent, reaching 0.67 Å and 1.0 Å, respectively. Even at these peaks, the profiles presented by the complexes were highly homogeneous. Furthermore, although it can be observed in Fig. 16a that NMT and the NMT-ligand complexes exhibited similar values in some regions, in the functionally important region of the protein, corresponding to the residues listed in Table S7 (Supplementary Information), the values for the complexes were consistently lower than those for the isolated NMT protein.

The deformability data in Fig. 16b show that the protein-ligand complexes exhibited similar profiles across the entire protein, with values ranging from approximately 0.15 Å to 0.30 Å. Nevertheless, peaks of higher deformability were observed, ranging from 0.5 Å to 0.8 Å, which were consistent with the regions that exhibited higher RMSF values, including the residues Phe14A (1.0 Å), Val81A (0.60 Å), Pro138A (0.58 Å), Pro182A (0.72 Å), Gly205A (0.58 Å), Phe232A (0.79 Å), Pro236A (0.69 Å), Gln238 (0.66 Å), Asn252A (0.60 Å), Asp287A (0.55 Å), Gly306A (0.50 Å), and Asp396A (0.54 Å). Besides, at these peaks, the highest deformability values were observed in the complexes with the reference drugs MTF and AmphoB.

**Fig. 17:**
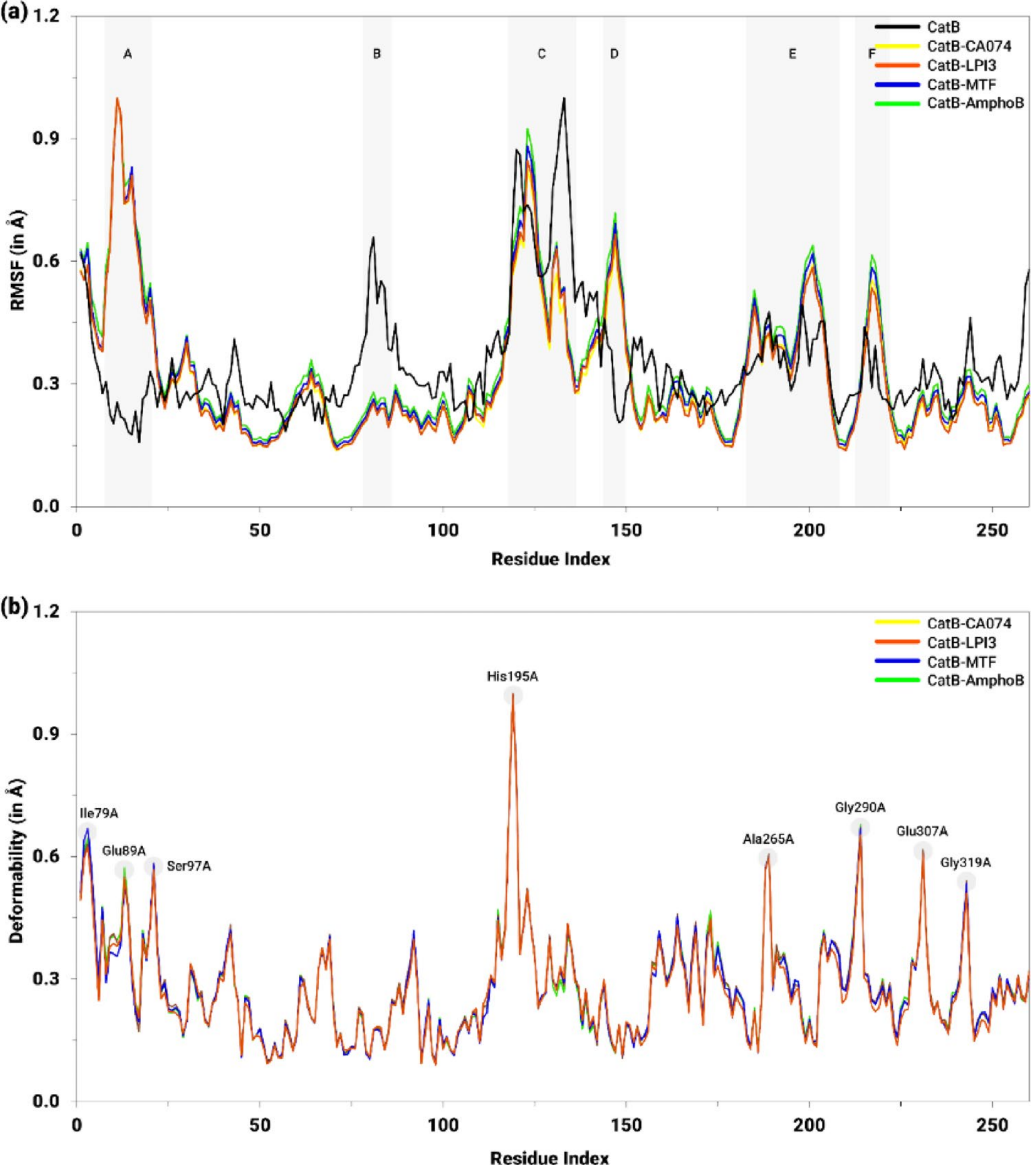
Flexibility (RMSF, in Å) and deformability (Å) profiles per residue for CatB in its isolated form and in complexes with the inhibitor CA074 (yellow), the derivative LPI3 (orange), and the drugs MTF (blue) and AmphoB (green). In **a**, the shaded regions (A–F) highlight segments with significant differences in flexibility. In **b**, the highlighted peaks indicate the residues with the highest deformability

 The flexibility and deformability profiles for the CatB-ligand complexes are shown in Fig. 17. With regard to flexibility, the NMA module applied to the isolated CatB protein yielded an RMSF ranging from 0.2 Å to 1.0 Å, with particular emphasis on the Cα conformational variations observed in regions B (Ser156A−Asp157A, 0.65 Å) and C (His195A−Gly201A, 0.87 Å; Ser206A−Asp211A, 1.0 Å) (Fig. 17a). The regions corresponding to the catalytic residues or those of the best pocket, described in Table S8 (Supplementary Information), exhibited moderate RMSF displacements, ranging from approximately 0.25 Å to 0.35 Å.

For the CatB-ligand complexes, although the ligands interacted with different regions of the protein, the NMA module highlighted that all evaluated complexes exhibited homogeneous behavior across the amino acid sequence, with predominantly low RMSF values, ranging from 0.15 Å to 0.35 Å (Fig. 17a). Among the ligands, the greatest fluctuations were observed in the CatB-AmphoB complex. In general, these complexes exhibited less residue displacement compared to isolated CatB. However, regions with pronounced conformational variations stood out, characterized by RMSF peaks, including the Ser78A−Pro93A (A), His195A−Tyr202A and Ser206A−Gly205A (C), Asp221A−Ile224A (D), Glu261A−Phe263A and Ser275A−Tyr278A (E), and Asn293A−Gly294A (F). In contrast, in the regions of the catalytic site and the residue of the best pocket, the values obtained were consistently lower than those observed in CatB without ligands.

Regarding deformability, it is observed that the complexes exhibit similar values across the protein residues, varying predominantly between 0.10 Å and 0.35 Å (Fig. 17b). The highest deformability value was observed at the His195A residue (1.0 Å), while intermediate values were observed at the Ile79A (0.64 Å), Glu89A (0.56 Å), Ser97A (0.58 Å), Ala265A (0.60 Å), Gly290A (0.67 Å), Glu307A (0.61 Å), and Gly319A (0.53 Å), which coincide with the protein segments that exhibited higher RMSF values. Although the profiles are generally similar, it is noted that the CatB-LPI3 complex exhibited slightly lower deformability values compared to the others. Additionally, the residues of the active site and the best pocket exhibited deformability values in the range of 0.15 Å and 0.30 Å.

**Fig. 18:**
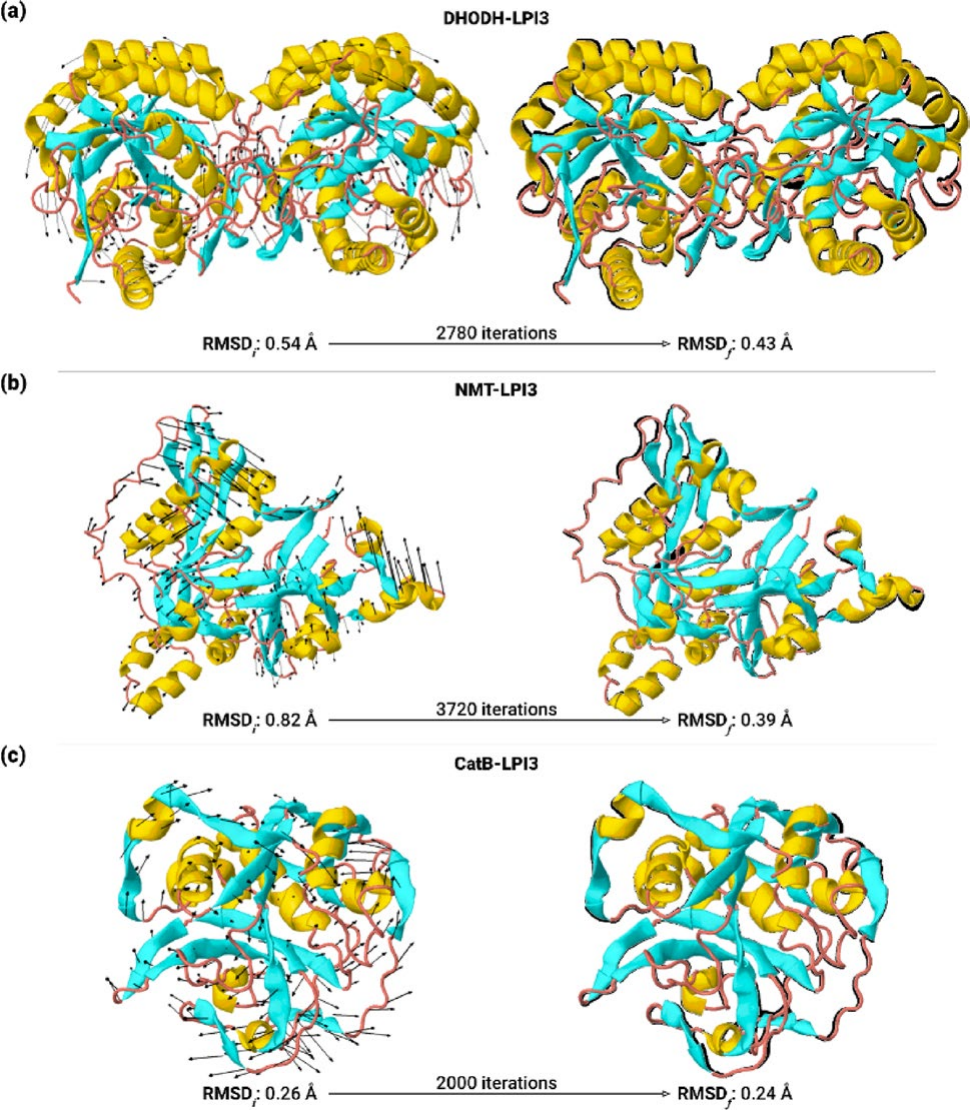
Convergence of the transformation trajectory for the protein-LPI3 complexes, measured by RMSD of the Cα atoms over the course of the iterations, for the proteins **a** DHODH, **b **NMT, and **c** CatB. The initial structure is represented with arrows indicating the direction of convergence of the α-helices (yellow), β-sheets (blue), and loops (red). The final structure is superimposed on the initial one (black) to facilitate visualization of conformational changes along the trajectory. RMSD_*i*_ refers to the initial conformational distance and RMSD_*f*_ to the final one

The structural convergence of the protein-LPI3 complexes is shown in Fig. 18. The convergence of the transformation trajectory can be observed in detailed in the Supplementary Information (Fig. S1–S3). The DHODH-LPI3 complex exhibited an initial structural deviation of 0.54 Å relative to the target protein (3TJX) (Fig. 18a). After 2,780 cycles, the complex converged to the target structure, exhibiting a Cα RMSD of approximately 0.43 Å. Comparison of the initial and final structures, based on their superposition, revealed that the protein maintained its overall structural arrangement without drastic conformational changes, exhibiting a subtle repositioning of the β-sheets.

A conformational deviation of 0.82 Å was observed in the zero-iteration cycle for the NMT-LPI3 complex relative to the NMT target structure (4CGL) (Fig. 18b). Over the course of the cycles, it was found that progressive structural convergence to the target protein was achieved after 3720 iterations, reaching a Cα RMSD of approximately 0.39 Å. Regarding the comparison between the initial and final structures, the global arrangement of the α-helix and β-sheets is maintained, accompanied by local structural adjustments in the loop regions, which exhibited variations in orientation and extension. For the CatB-LPI3 complex, shown in Fig. 18c, the complex exhibited an initial structural deviation of 0.27 Å relative to the crystallized structure of CatB (3MOR). Subsequently, the complex exhibited high structural convergence after 2000 iterations, evidenced by a Cα RMSD of 0.24 Å. Structurally, the global arrangement of the protein after the simulations was preserved, showing discrete variations in the loop regions.

### MPO analysis and ADMET prediction

#### Physicochemical properties

**Fig. 19:**
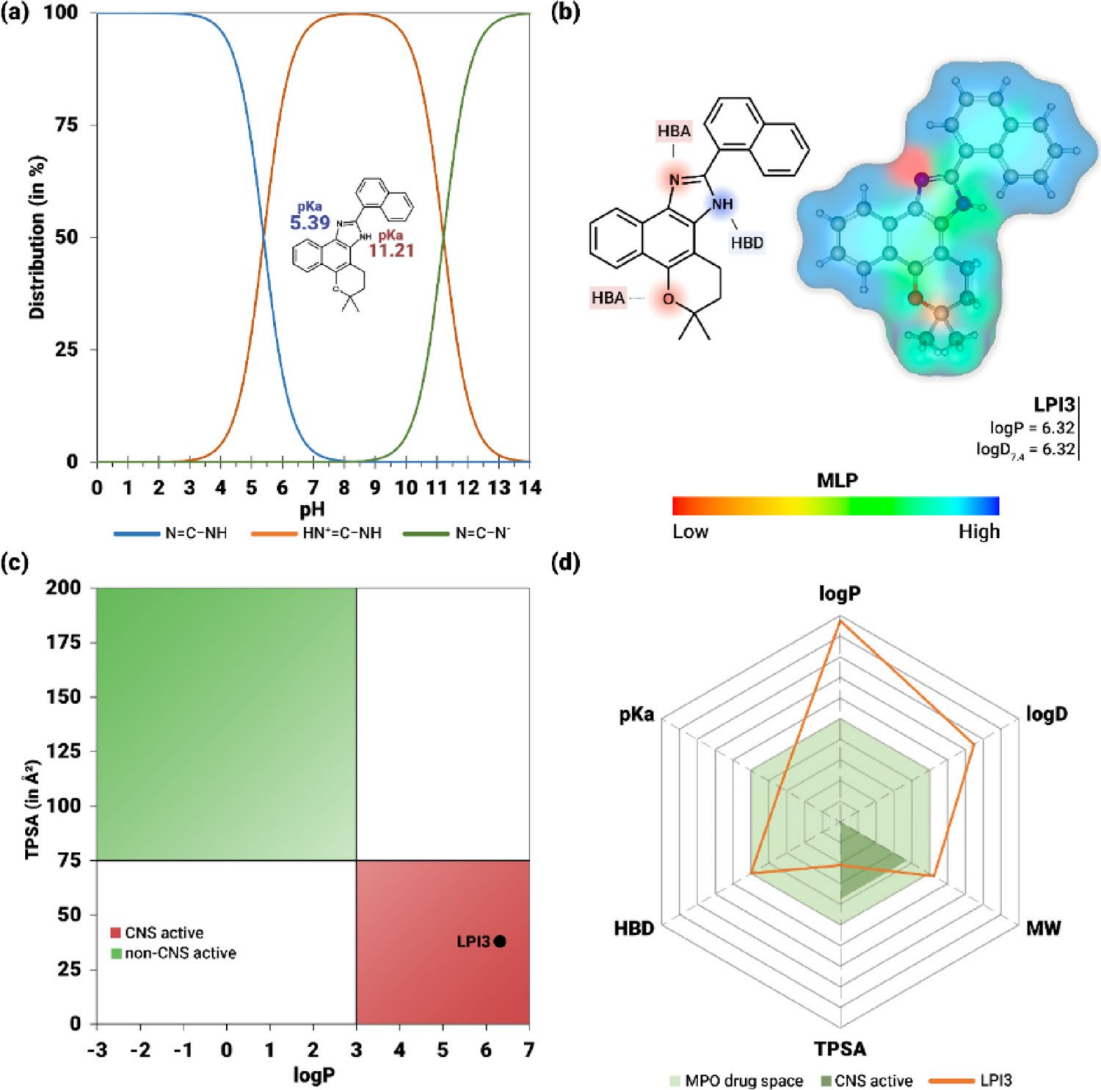
Physicochemical properties and drug-likeness of the lead compound LPI3. In **a**, the distribution of protic, neutral, and anionic species of compound LPI3 as a function of pH is shown. In **b**, a molecular lipophilicity potential (MLP) map highlighting hydrophobic and hydrophilic regions, as well as hydrogen bond donor (HBD) and acceptor (HBA) sites. In **c**, a graph of susceptibility to activity in the central nervous system, based on the partition coefficient (logP) and the topological polar surface area (TPSA). In **d**, a multi-parameter optimization (MPO) radar plot used to estimate properties related to the ADMET profile

Figure 19; Table 4 present the physicochemical properties and drug-likeness of compound LPI3. The compound has a molecular weight of 378.48 g/mol and a highly rigid structure, consisting of six aromatic rings and only one rotatable bond (Table 4). Additionally, it exhibits low hydrogen bonding potential (HBA = 2 and HBD = 1) and a TPSA of 37.91 Å^2^. In the microspecies distribution plot, it is observed that the imidazole HBA nitrogen (= N) acts as a basic center, with a pKa of 5.39, reaching the equivalence point with the protic species (− NH^+^) at the corresponding pH. Meanwhile, the imidazole HBD nitrogen (− NH) exhibits acidic character (pKa = 11.21), with an equivalence point at pH 11.21, corresponding to the formation of the anionic species (− N^-^). Furthermore, the neutral form of the compound predominates at physiological pH (approximately 7.4), with an estimated fraction of 99.01% (Fig. 19a).

In relation to molecular lipophilicity potential (MLP), represented by the surface in Fig. 19b, LPI3 exhibits a predominance of lipophilic regions (green and blue spectra), distributed mainly over the aromatic rings. In contrast, polar regions (yellow and red spectra) are limited and are located mainly on the HBA nitrogen of the imidazole moiety and on the asymmetric carbon of the dihydropyran ring. The lipophilic profile of this compound is consistent with the high values of intrinsic lipophilicity (logP = 6.32) and in buffer at pH 7.4 (logD = 6.32). Furthermore, the physicochemical properties of LPI3 resulted in an estimated oral bioavailability index of 55% (ABS = 0.55) (Table 4).

**Table 4:** Physicochemical properties and drug-likeness assessment of the β-Lapachone derivative LPI3

Property		LPI3	Max. limit
Physicochemical properties	Molecular weight (g/mol)	378.17	360.00
	Aromatic rings	6	3
	Rotatable bonds	1	10
	H-Bond acceptors	2	10
	H-Bond donors	1	1
	TPSA (Å^2^)	37.91	90.00
	pKa most basic	5.39	8.0
	logP	5.848	3.0
	logD	4.492	2.0
Drug-likeness	ABS	0.55	−
	Brenk alerts	0 alerts	−
	Lipinski’s rule	Accepted	−
	Pfizer rule	2 alerts: logP > 3, TPSA < 75 Å^2^	−
	Golden triangle	Accepted	−
	QED score	0.355	> 0.5
	MPO score	3.6	> 3.0 − 4.0

#### Drug-likeness evaluation

The drug-likeness assessment indicated no structural alerts (Brenk alerts = 0) and showed that the compound satisfies the criteria established by Lipinski’s rule and the Golden Triangle model (Table 4). However, two violations were identified according to the Pfizer rule, related to high lipophilicity (logP > 3) and a small topological polar surface area (TPSA < 75 Å^2^). The relationship between these two properties, shown in Fig. 18c, demonstrates that the compound is distributed within a physicochemical space of likely CNS activity. Consequently, the drug similarity index, on a scale of 0 to 1, was 0.355, based on the QED scoring function. It should also be noted that the bioavailability radar chart, shown in Fig. 18d, illustrates the distribution of the parameters that make up the MPO score. For compound LPI3, the obtained value (MPO = 3.6) falls within the range considered acceptable (3–4), with a strong contribution from the penalty for logP > 3.0, logD > 2.0, and MW > 360 g/mol (Table 4).

#### Pharmacokinetics descriptors

The prediction of ADMET descriptors (Table 5) indicated that LPI3 has low water solubility (−2.892 log mol/L) and low skin permeability (−2.735 log Kp in cm/h) and in Caco-2 cells (−0.446 log Papp in 10⁻^6^ cm/s). However, an intestinal absorption rate of 79.255% was observed, associated with moderate Caco-2 permeability. Furthermore, the compound showed a high probability of acting as a substrate and/or inhibitor of Pgp, reducing the passive cellular efflux of co-administered substances. Regarding distribution parameters, it is estimated that the compound has a moderate Vdss (0.088 log L/kg) and an equally moderate plasma free fraction (33.2%). Potential for crossing the blood-brain barrier (0.407 log BB) was also observed, as well as possible activity in the CNS (−0.247 log PS), consistent with the profile previously observed in Fig. 18c.

**Table 5:** Predicted absorption, distribution, metabolism, excretion, and toxicity descriptors for LPI3

Property		Predicted value	Property		Predicted value
Absorption	Water solubility (log mol/L)	−2.892	Metabolism	CYP2C19 inhibitor	Yes
	Caco-2 permeability (log P_app_ in 10^− 6^ cm/s)	−0.446		CYP2C9 inhibitor	Yes
	HIA (%)	79.255		CYP2D6 inhibitor	No
	Skin Permeability (log Kp)	−2.735		CYP3A4 inhibitor	No
	Pgp substrate	Yes	Excretion	Total Clearance (log ml/min/kg)	0.989
	Pgp I inhibitor	Yes		Renal OCT2 substrate	Yes
	Pgp II inhibitor	Yes	Toxicity	Human max. dose (log mg/kg/day)	0.406
Distribution	VDss (log L/kg)	0.088		Oral Rat Acute Toxicity (LD_50_) (mol/kg)	2.37
	Fraction unbound (Fu)	0.332		Oral Rat Chronic Toxicity (LOAEL) (log mg/kg_bw/day)	0.829
	BBB permeability (log BB)	0.407		AMES toxicity	Yes
	CNS permeability (log PS)	−0.247		Hepatotoxicity	No
Metabolism	CYP2D6 substrate	Yes		Skin Sensitization	No
	CYP3A4 substrate	Yes		hERG I inhibitor	No
	CYP1A2 inhibitor	Yes		hERG II inhibitor	Yes

#### Site of metabolism prediction

**Fig. 20:**
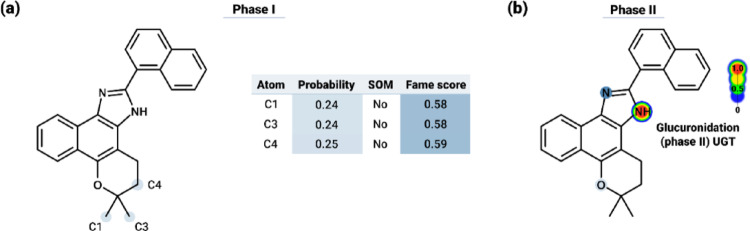
Predicted metabolic sites for the LPI3 derivative. **a** Three possible metabolic sites were identified; however, they did not score high enough to be considered relevant Phase I metabolism sites. **b** The secondary amine group is indicated as a potential site for conjugation, with the formation of a glucuronide conjugate mediated by UDP-glucuronosyltransferases (UGT), characterizing a Phase II metabolism reaction

In terms of metabolic prediction, the compound was found to act as an inhibitor and substrate for different cytochrome P450 isoforms (Table 5). Specifically, the compound was predicted to be an inhibitor of the CYP1A2, CYP2C9, and CYP2C19 isoforms, whereas for the CYP2D6 and CYP3A4 isoforms, LPI3 was classified exclusively as a substrate. In this sense, the analysis of potential Phase I metabolism sites, presented in Fig. 20a, revealed preferential regions of biotransformation distributed throughout the ligand structure, notably at atoms C1 (probability = 0.24; Fame score = 0.58), C3 (probability = 0.24; FAME score = 0.58) and C4 (probability = 0.25; FAME score = 0.59). Despite this, none of the atoms were classified as positive SOM. Additionally, it is observed that the nitrogen and the secondary amine of the imidazole moiety constitute Phase II metabolism sites, enabling the formation of a glucuronide conjugate via UGT (UDP-glucuronosyltransferase) for renal excretion (Fig. 20b). In this context, regarding excretion prediction, LPI3 exhibited a predicted total clearance of 0.989 log ml/min/kg and was identified as a substrate of the OCT2 transporter (Table 5).

#### Toxicity prediction

With regard to toxicity prediction, the compound had a maximum tolerable dose for humans of 0.406 log mg/kg/day. Acute oral toxicity in rats (LD_50_) was estimated at 2.37 mol/kg, while chronic toxicity (LOAEL) was 0.829 log mg/kg_bw/day (Table 5). Additionally, the compound exhibited mutagenic potential, but no evidence of hepatotoxicity or skin sensitization was observed (Table 5).

**Fig. 21:**
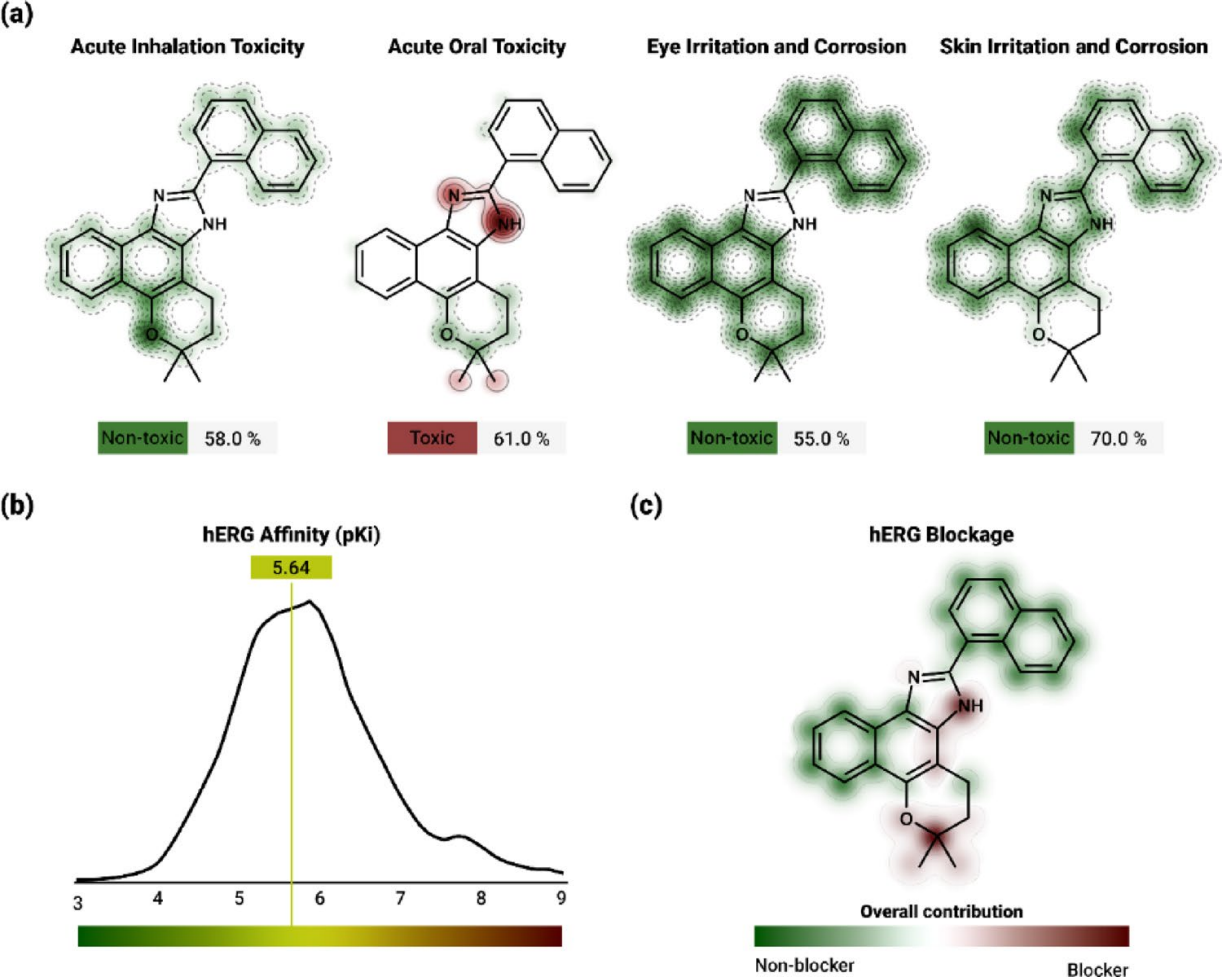
Predicted acute toxicity profile for compound LPI3. In **a**, the structural contribution and probability of acute toxicity for different exposure routes are shown. In **b**, the non-intuitive structure-activity relationship related to affinity for the hERG channel is shown. In **c**, the molecular contributions associated with the potential for hERG channel blockade are shown

Figure 21 presents the predictive assessment of acute toxicity and cardiotoxicity. Regarding acute toxicity, the model indicated an absence of inhalation toxicity (58% classified as non-toxic), as well as an absence of eye irritation and corrosion (55% non-toxic) and skin irritation (70% non-toxic). In contrast, it indicated acute oral toxicity (61%), associated mainly with the pyridinium nitrogen and the secondary amine of the imidazole moiety, as well as the methyl groups attached to the dihydropyran ring (Fig. 21a). Regarding cardiotoxicity, the assessment based on affinity for the hERG ion channel indicated moderate intrinsic risk, with an estimated pKi of 5.64 and a 57% probability of interaction (Fig. 21b). In this case, the compound was classified as a moderate hERG II inhibitor, with structural contributions from the secondary amine of the imidazole moiety and the quaternary carbon of the dihydropyran ring.

## Discussion

Initially, pocket prediction revealed substantial structural differences between the molecular targets. In this case, the *Leishmania*-specific targets, DHODH and NMT, exhibited large pockets with diverse chemical compositions, suggesting greater potential for multiple modes of interaction. In contrast, CatB exhibited a smaller best pocket and a more complex functional organization, as the catalytic site was distributed across two adjacent cavities, slightly separated from the main pocket. This structural feature may explain, to some extent, the greater variability observed in the binding results for this enzyme, particularly with regard to the RMSD values.

Specifically, DHODH is an enzyme associated with the inner mitochondrial membrane and is essential for *de novo* pyrimidine biosynthesis, involving electron transfer to the cofactor FMN (Arakaki et al. 2008). Given that the inner mitochondrial membrane is highly hydrophobic and has low permeability to polar molecules, this environment imposes structural constraints on both the enzyme and potential interactions with the evaluated ligands. Although the active site exhibited a mixed composition of residues, the surface of the best pocket showed a predominance of nonpolar residues, favoring the establishment of hydrophobic interactions (Bogunia and Makowski 2020). On the other hand, the interior of this cavity was composed of polar and charged residues, favoring the formation of hydrogen bonds, as shown in the interaction analysis.

NMT, however, is distributed equally in the cytosol and the plasma membrane (Panethymitaki et al. 2006). The cytosol is a predominantly aqueous medium containing ions and organic components, while the plasma membrane is a semipermeable barrier consisting of a bilayer of phospholipids and proteins (Feher 2012). Consequently, proteins located in these different environments possess an amphipathic character, with a heterogeneous distribution of polar and nonpolar residues. Accordingly, it was observed that the best pocket of this protein followed this profile, since, although it was composed mainly of nonpolar residues, the entrance to this region, where most of the active site residues and the evaluated ligands were located, was also formed by polar residues, suggesting a functional role in selectivity and molecular recognition (Persch et al. 2015). Therefore, this arrangement may facilitate access by compounds from the aqueous medium, while simultaneously stabilizing the complex within the pocket through hydrophobic interactions and π-stacking, reflecting a structural adaptation to the hybrid environment in which it is embedded, as observed in this study.

As for CatB, it is a cysteine protease located in the lysosome, a highly specialized intracellular compartment that acts as a center for cellular degradation and metabolism, characterized by an acidic pH of approximately 4.5 to 5.0 (Yang and Wang 2021). Unlike neutral compartments, such as the cytosol, the lysosomal environment promotes the protonation of ionizable residues, resulting in changes in intramolecular interactions and protein conformational stability. As a result, lysosomal proteins have a higher proportion of polar and charged residues, capable of maintaining stable interactions and performing catalytic functions at acidic pH (Chen et al. 2025b). The results obtained from the pocket prediction for CatB corroborated these characteristics, as the protein exhibited a predominance of polar and ionizable residues, particularly in regions that comprise or surround the catalytic site, suggesting a profile favorable to acid-base catalysis and ligand stabilization.

The redocking step for the co-crystallized inhibitors demonstrated satisfactory reproduction of the experimental poses, with RMSD values within acceptable limits, confirming the robustness of the protocol used (Shityakov and Foerster 2014). In general, the interaction profiles of the protein-inhibitor complexes were consistent with the physicochemical characteristics of each binding site. Similar results were reported for DHODH, NMT, and CatB, as described by Razzaghi-Asl and Hashemi (Razzaghi-Asl and Hashemi 2022), Roberto et al. (Roberto et al. 2025), and Abdelfattah et al. (Abdelfattah et al. 2021), respectively.

As regards conformational consistency, the RMSD values remained below 2.0 Å, indicating high reproducibility of the conformations and adequate structural stability of the complexes formed, with differences depending on both the ligand and the molecular target. Notably, the β-lapachone derivatives exhibited lower RMSD values compared to the reference drugs and co-crystallized inhibitors, suggesting greater conformational predictability and better accommodation of the compounds within the cavity. This pattern may be associated with the structural characteristics of the naphthoquinone core (composed of naphthalene and dihydropyran) present in the β-lapachone derivatives, which imparts greater rigidity to the molecules (Saxena et al. 2020). Similar results were observed by De Molfetta et al. (De Molfetta et al. 2009), Venkatesan, Shukla and Dubey (Venkatesan et al. 2010), Vera et al. (Vera et al. 2017)d rez-Pertejo et al. (Pérez-Pertejo et al. 2019), particularly in complexes involving quinone compounds and molecular targets from protozoa.

These derivatives exhibited substantially more favorable affinity energy values compared to the controls, indicating greater theoretical affinity and suggesting that these compounds exhibit greater selectivity for *Leishmania* targets (Kitchen et al. 2004). Chen et al. (Chen et al. 2016) suggest that this behavior occurs because polar functional groups, such as those present in these derivatives, can act as hydrogen bond donors and acceptors, which favors directional interactions with key residues in the active site. In this case, the pattern of mixed interactions with hydrophobic and polar residues may be associated with greater stability of protein-ligand complexes, as also observed by Raschka et al. (Raschka et al. 2018).

It is also worth noting a structural similarity between portions of the evaluated derivatives and chemical fragments present in ligands described in the literature as having antiprotozoal activity. The naphthalene moiety has been widely associated with leishmanicidal and trypanocidal activity, as observed in the compounds from the studies by Costa et al. (Costa et al. 2017) and Peixoto et al. (Peixoto et al. 2021). Regarding the imidazole moiety, this constitutes a recurring structural motif in antiparasitic compounds, frequently involved in interactions with catalytic residues of molecular targets, as demonstrated in trypanothione reductase inhibitors described by Pandey et al. (Pandey et al. 2015). This structural correspondence may contribute not only to better accommodation of the compounds within the pocket of the protozoan proteins but may also explain the more favorable affinity energy values obtained in this study.

In this regard, the RMSD×EA relationship, represented by the quadrant diagrams, enabled a more objective analysis of each ligand’s selectivity, where compounds distributed in Q_3_ were considered the most promising, as they combined greater conformational stability (low RMSD) and high affinity (low EA) for the targets. Therefore, the predominance of these derivatives in this quadrant, particularly LPI3, reinforces their potential as candidates for effective inhibitors.

On the whole, the analysis of interactions indicated that the stability of the complexes is related to the complementarity between the ligands and the protein pocket environment. This pattern is widely described for inhibitors of *Leishmania* targets, in which hydrophobic interactions promote initial anchoring, while hydrogen bonds with catalytic residues contribute to molecular recognition specificity, as reported by De Oliveira et al. (De Oliveira et al. 2025). However, conformational dynamics analysis demonstrated that the stability of these complexes does not depend solely on the presence of these interactions, but also on their persistence throughout time, corroborating studies that highlight variability between poses obtained by docking and the actual dynamic behavior of the complexes (Sakano et al. 2016; Gómez Borrego and Torrent Burgas 2025). Additionally, it was observed that the active site residues of both proteins exhibited greater structural stability throughout the simulations. Notably, the fluctuation and deformability profiles were highly similar, even when ligands bound to different regions of the protein, as observed in the CatB-ligand complexes.

In this context, it was found that the compound LPI3 bound to the largest predicted pocket of this protein, while the reference drugs interacted with the cavity that encompasses the active site. This result suggests that the larger-volume pocket does not necessarily correspond to a functionally relevant region of the enzyme, but may represent an allosteric region, as identified by Costa et al. (Costa et al. 2010) for the action of heparin in inhibiting this protein and by the study conducted by Novinec et al. (Novinec et al. 2010) with cathepsin K. The preferential occupation of this cavity by the derivative may be related to greater structural accessibility, rather than specific recognition of the catalytic site. Despite this difference in the binding site, no significant variations in the stability of the complexes were observed, indicating that the interaction of the derivative with the alternative cavity is structurally stable, even if potentially non-productive from a catalytic standpoint. This behavior may also indicate greater selectivity of β-lapachone derivatives for *Leishmania* molecular targets, compared to those of other trypanosomatids. Therefore, enzyme inhibition assays are essential to determine whether this interaction exerts any functional effect, either through direct inhibition or via possible allosteric mechanisms.

Under this perspective, lapachol-derived compounds are widely described in the literature for their antiprotozoal activity, which is commonly associated with the generation of reactive oxygen species and the modulation of cellular redox processes (Ventura Pinto and Lisboa De Castro 2009; Bombaça et al. 2019). Although these mechanisms are not directly related to molecular docking, they reinforce the biological potential of the compounds, as well as the relevance of the results obtained. In relation to this, in vitro studies have indicated that these compounds are capable of significantly reducing the viability of promastigote and amastigote forms of *Leishmania* and other protozoa (Costa et al. 2017; Araújo et al. 2019; Pertino et al. 2020). Similarly, Araújo et al. (Araújo et al. 2019) reported a reduction in parasite burden in vivo, accompanied by an improvement in clinical signs in experimental models.

Although the LPI3 compound exhibited high affinity and conformational stability, as discussed earlier, from a pharmacological standpoint these characteristics alone do not guarantee therapeutic efficacy, since the compound must reach the site of action at an adequate concentration, cross biological barriers, resist early metabolism, and exhibit no significant toxicity (Van De Waterbeemd and Gifford 2003). Therefore, the prediction of ADMET properties serves as a physiological filter for the activity of this derivative in silico. In this context, the physicochemical parameters of the compound play a central role in determining this behavior, given that they directly influence processes ranging from absorption to excretion. The parameters of the LPI3 derivative indicate a profile compatible with good pharmacological characteristics, as evidenced by the MPO analysis, suggesting an adequate balance between properties such as lipophilicity, polarity, and hydrogen-bonding potential, fundamental aspects for performance in the body (Wager et al. 2010b).

Considering absorption, the descriptors indicated that, despite its low water solubility, the compound exhibits good intestinal absorption and low permeability in Caco-2 cells. Physiologically, this profile can be explained by the ability of lipophilic compounds to cross the enterocyte membrane via passive diffusion, even when their solubility is limited (Lipinski et al. 1997). However, the low permeability observed in Caco-2 models suggests that transcellular diffusion may not be fully efficient, possibly due to the action of efflux transporters, such as Pgp, or to limitations imposed by the compound’s structural rigidity, reducing the amount effectively absorbed (Artursson et al. 2001). Generally, high lipophilicity favors interaction with biological membranes and may result in a larger volume of distribution, allowing the compound to reach intracellular compartments (Kerns and Di 2008). This characteristic is important in the context of leishmaniasis, given that therapeutic efficacy depends on the drug’s ability to reach intracellular environments where the parasite is located.

Regarding metabolism, the presence of the imidazole group suggests, based on the literature, a susceptibility to hepatic biotransformation, primarily by cytochrome P450 isoforms. Guengerich (Guengerich 2008) describes that compounds containing nitrogen-containing heterocycles, such as the imidazole moiety, are frequently associated with the modulation of these enzymes. However, in the present study, no evidence of a significant influence of the imidazole group on CYP450-mediated biotransformation was observed. This result suggests that, for the evaluated derivative, the imidazole group does not play a decisive role in hepatic metabolism, which may positively affect the predictability of bioavailability and reduce the potential for drug interactions (Zanger and Schwab 2013).

These metabolic transformations increase the polarity of the compounds to facilitate their hepatic and renal clearance (Zanger and Schwab 2013). Given the initial lipophilic profile of LPI3, it is likely that its elimination occurs primarily after biotransformation via the aforementioned pathways. This process may prolong the systemic half-life of this derivative in the body, which may be beneficial by allowing for a longer interaction time between the compound and the molecular target. Conversely, this same characteristic may increase the risk of accumulation and adverse effects, especially during prolonged treatments, as occurs with the use of reference drugs.

Finally, regarding toxicity, it is observed that the toxicity profile of LPI3 is influenced by the balance between bioactivation and detoxification processes. In contrast, the absence of hepatotoxicity suggests that detoxification mechanisms, especially those associated with Phase II reactions, such as UGT-mediated glucuronidation, may be effective in increasing polarity and facilitating excretion (Rowland et al. 2013). Furthermore, acute toxicity analysis demonstrated the absence of relevant effects via systemic routes, such as inhalation and dermal exposure, while oral toxicity was associated with specific functional groups. Similar results have been reported in in silico studies and confirmed in in vitro models, in which modifications to nitrogen-containing heterocycles directly impact the toxicological profile (Rahman et al. 2021; Kumar et al. 2025). Another important factor relates to the prediction of cardiotoxicity, which indicated a moderate risk. This characteristic may be associated with the compound’s potential interaction with the hERG channel, which is involved in drug-induced arrhythmias (Vandenberg et al. 2012). Thus, although LPI3 exhibits favorable pharmacokinetic properties, its toxicological profile underscores the need for in vitro and in vivo analyses to assess its biological activity.

## Conclusion

The results obtained in this study demonstrated that the integrated computational approach employed was effective in evaluating the antileishmanial potential of β-lapachone derivatives, enabling a comprehensive characterization of structural, pharmacokinetic, and molecular interaction properties with essential targets of *Leishmania *spp. Among the compounds evaluated, the LPI3 derivative stood out as the most promising candidate, exhibiting favorable physicochemical and pharmacokinetic profiles compatible with drug-likeness and safety criteria. Furthermore, docking and NMA-based conformational dynamics analysis indicated that this compound establishes stable and energetically favorable interactions with molecular targets, exhibiting superior performance compared to reference drugs, especially with proteins specific to the *Leishmania* genus, suggesting greater potential for selectivity and biological efficacy.

These findings reinforce the potential of LPI3 as a candidate for an antileishmanial drug and highlight the importance of in silico strategies in the screening and optimization of bioactive compounds. In this context, these results contribute to the development of new treatments, as well as to the advancement of rational drug design for the treatment of leishmaniasis. Nevertheless, it should be noted that computational predictions have specific limitations, as they do not fully account for the complexity of biological systems. Therefore, the results must be interpreted appropriately, and it is essential to conduct in vitro and in vivo experimental studies to validate the antileishmanial activity and confirm the compound’s pharmacokinetic and toxicological profile. Finally, future studies should explore the biological validation of the lead compound, in addition to applying complementary approaches, such as more robust molecular dynamics simulations and structural optimization strategies, to improve the efficacy, selectivity, and safety of the derivative.

## Supplementary Information

Below is the link to the electronic supplementary material.

Supplementary Material 1

## Data Availability

All the data generated or analyzed during this study are included in this published article.
