# Supplementary material for: Targeting of Leishmania proteins by β-Lapachone derivatives reveals a promising multitarget candidate
Source: Arch Microbiol. 2026 Jun 6;208(8):428. doi: 10.1007/s00203-026-04989-1 (PMC13242429; doi:10.1007/s00203-026-04989-1)
Supplement: Supplementary file 1 — Supplementary Material 1 [file 203_2026_4989_MOESM1_ESM.docx]

Targeting of *Leishmania* Proteins by β-Lapachone Derivatives Reveals a Promising Multitarget Candidate

Archives of Microbiology

Laércio Mariano-Fernandes^a^, Jacilene Silva^b^, Matheus Nunes da Rocha^c^, Márcia Machado Marinho^c^, Emmanuel Silva Marinho^a,c,*^

^a^ *Postgraduate Program in Veterinary Sciences, State University of Ceará, Fortaleza, Ceará, Brazil*

^b^ *Department of Biological Chemistry, Regional University of Cariri, Crato, Ceará, Brazil*

^c^ *Postgraduate Program in Natural Sciences – PPGCN, State University of Ceará, Fortaleza, Brazil*

* **Corresponding author at:** Bioprospecting and Natural Resource Monitoring Laboratory, State University of Ceará, Av. Dr. Silas Munguba, 1700 - Itaperi Campus, Fortaleza, CE, CEP: 60.714.903, Brazil

*E-mail address:* [emmanuel.marinho@uece.br](mailto:emmanuel.marinho@uece.br) (E.S. Marinho).

**ORCID of the authors**

L. Mariano-Fernandes: 0000-0003-2130-804X

J. Silva: 0000-0001-5008-858X

M.N. da Rocha: 0000-0002-0929-4565

M.M. Marinho: 0000-0002-7640-2220

E.S. Marinho: 0000-0002-4774-8775

1. **Molecular Docking Simulations for β-Lapachone-derived Compounds**

**Table S1. RMSD of Protein-Ligand Complexes**

| **Ligand** | **DHODH (3MHU)**  **RMSD (Å)** | **NMT (2WUU)**  **RMSD (Å)** | **CatB (3HHI)**  **RMSD (Å)** |
| --- | --- | --- | --- |
| **LPI1** | 1.413 | 1.324 | 1.603 |
| **LPI2** | 1.070 | 0.888 | 1.226 |
| **LPI3** | 0.868 | 0.786 | 0.909 |
| **LPI4** | 0.779 | 0.873 | 0.798 |
| **LPI5** | 1.077 | 0.804 | 1.598 |
| **LPI6** | 1.215 | 0.786 | 0.753 |
| **LPI7** | 1.630 | 1.529 | 1.653 |
| **MTF*** | 1.219 | 1.378 | 1.433 |
| **AmphoB*** | 1.997 | 1.441 | 1.401 |
| **Inhibitor**** | 1.913 | 1.483 | 1.865 |

*Control ** EJZ (DHODH), NHW (NMT) and CA074 (CatB)

**Table S2. Affinity Energy of Protein-Ligand Complexes**

| **Ligand** | **DHODH (3MHU)**  **E_A_ (kcal/mol)** | **NMT (2WUU)**  **E_A_ (kcal/mol)** | **CatB (3HHI)**  **E_A_ (kcal/mol)** |
| --- | --- | --- | --- |
| **LPI1** | -8.4 | -9.8 | -8.0 |
| **LPI2** | -9.2 | -10.7 | -7.9 |
| **LPI3** | -9.4 | -12.2 | -8.9 |
| **LPI4** | -8.7 | -10.5 | -8.3 |
| **LPI5** | -9.3 | -10.9 | -7.5 |
| **LPI6** | -8.6 | -10.8 | -7.9 |
| **LPI7** | -9.1 | -11.4 | -8.5 |
| **MTF*** | -5.7 | -7.2 | -4.4 |
| **AmphoB*** | -7.7 | -8.2 | -7.3 |
| **Inhibitor**** | -7.4 | -8.9 | -5.7 |

*Control ** EJZ (DHODH), NHW (NMT) and CA074 (CatB)

1. **Interaction Analysis Between β-lapachone-derived compounds and Protein Structure**

**Table S3. Interaction types between ligands and Dihydroorotate dehydrogenase**

| **Ligand** | **Interaction Type** | **Residue (Distance in Å)** |
| --- | --- | --- |
| **LPI1** | Hydrophobic | Met70A (3.74), Lys215A (3.62) |
|  | H-Bond | Ser196A (2.90), Asn199A (3.18) |
| **LPI2** | Hydrophobic | Ser69A (3.83), Gln139A (3.53), Asp171B (3.59), Phe172B (3.50), Ala173B (3.77) |
|  | H-Bond | Asn199A (2.8) |
| **LPI4** | Hydrophobic | Gln139A (3.59), Asp171B (3.88), Phe172B (3.42), Ala173B (3.74) |
|  | H-Bond | Asn199A (2.88) |
| **LPI5** | Hydrophobic | Gln139A (3.62), Tyr142A (3.55), Pro167A (3.76), Asp171B (3.80), Phe172B (3.71), Ala173B (3.78) |
|  | H-Bond | Asn199A (2.96) |
| **LPI6** | Hydrophobic | Ser69A (3.62), Asp171B (3.59) |
|  | H-Bond | Asn199A (2.76) |
| **LPI7** | Hydrophobic | Met70A (3.66), Gln139A (3.75), Tyr142A (3.78), Asp171B (3.67) |
|  | H-Bond | Asn199A (2.84), Lys215A (3.49) |

**Table S4. Interaction types between ligands and *N*-myristoyltransferase**

| **Ligand** | **Interaction Type** | **Residue (Distance in Å)** |
| --- | --- | --- |
| **LPI1** | Hydrophobic | Tyr217A (3.51), Tyr217A (3.76) |
|  | H-Bond | Tyr345A (1.98) |
|  | π-Stacking | Tyr217A (3.85), Tyr217A (4.75) |
| **LPI2** | Hydrophobic | Val81A (3.40), Phe90A (3.96), Tyr217A (3.35), Tyr217A (3.67), Tyr217A (3.84) |
|  | π-Stacking | Tyr217A (3.97) |
| **LPI4** | Hydrophobic | Tyr80A (4.00), Val81A (3.54) |
|  | H-Bond | Asn167A (2.42) |
|  | π-Stacking | Tyr217A (4.19) |
| **LPI5** | Hydrophobic | Val81A (3.72), Val206A (3.37), Tyr217A (3.99) |
|  | H-Bond | Gly205A (2.55) |
|  | π-Stacking | Tyr217A (3.88) |
| **LPI6** | Hydrophobic | Val81A (3.49), Val206A (3.39) |
|  | H-bond | Gly205A (2.69) |
|  | π-Stacking | Tyr217A (3.85) |
| **LPI7** | Hydrophobic | Val81A (3.76), Tyr217A (3.53), His219A (3.79), Phe232A (3.89), Ile328A (3.89), Tyr345A (3.51) |
|  | H-Bond | Tyr345A (2.20) |
|  | π-Stacking | Tyr217A (4.02) |

**Table S5. Interaction types between ligands and Cathepsin B**

| **Ligand** | **Interaction Type** | **Residue (Distance in Å)** |
| --- | --- | --- |
| **LPI1** | Hydrophobic | Gln112A (3.4), Ala114A (3.52), Val125A (3.53), Ala148A (3.24), Tyr187A (3.72), Tyr187A (3.4) |
|  | H-Bond | Asp115A (2.33), Ser129A (2.3) |
| **LPI2** | Hydrophobic | Val125A (3.49), Ala128A (3.5), Ala148A (3.67) |
|  | H-Bond | Asp182A (2.15) |
| **LPI4** | Hydrophobic | Val125A (3.41), Ala148A (3.53), Pro186A (3.66), Tyr187A (3.77) |
|  | H-Bond | Val125A (2.2), Ser129A (2.81), Asp182A (2.24) |
| **LPI5** | Hydrophobic | Gln112A (3.79), Val125A (3.69) |
|  | π-Stacking | His191A (5.07) |
| **LPI6** | Hydrophobic | Pro186A (3.48), Tyr187A (3.57) |
|  | H-Bond | Asp182A (2.6), Tyr187A (2.95) |
| **LPI7** | Hydrophobic | Asp115A (3.7), Ala128A (3.75), Ala148A (3.56), Pro186A (3.26) |
|  | H-Bond | Asp182A (2.08) |

1. **NMA-based Molecular Dynamics**

**Table S6. Active Site Residues of DHODH Characterized by Molecular Dynamics Simulation**

| **Residue Index** | **Atom** | **Amino Acid** |
| --- | --- | --- |
| 47 | Cα | Lys44A |
| 71 | Cα | Asn68A |
| 73 | Cα | Met70A |
| 74 | Cα | Gly71A |
| 75 | Cα | Leu72A |
| 103 | Cα | Ser100A |
| 104 | Cα | Gly101A |
| 105 | Cα | Leu102A |
| 110 | Cα | Asn107A |
| 131 | Cα | Asn128A |
| 132 | Cα | Leu129A |
| 133 | Cα | Ser130A |
| 134 | Cα | Cys131A |
| 135 | C | Cys131A |
| 136 | N | Gln139A |
| 137 | Cα | Gln139A |
| 138 | Cα | Val140A |
| 148 | Cα | Cys150A |
| 193 | Cα | Asn195A |
| 194 | Cα | Ser196A |

**Table S7. Active Site Residues of NMT Characterized by Molecular Dynamics Simulation**

| **Residue Index** | **Atom** | **Amino Acid** |
| --- | --- | --- |
| 71 | Cα | Tyr80A |
| 72 | Cα | Val81A |
| 73 | Cα | Glu82A |
| 74 | C | Glu82A |
| 78 | Cα | Phe88A |
| 80 | Cα | Phe90A |
| 82 | Cα | Tyr92A |
| 157 | Cα | Asn167A |
| 193 | Cα | Thr203A |
| 207 | Cα | Tyr217A |
| 209 | Cα | Tyr219A |
| 222 | Cα | Phe232A |
| 316 | Cα | Tyr326A |
| 318 | Cα | Ile328A |
| 320 | Cα | Ser330A |
| 327 | Cα | Leu341A |
| 329 | Cα | Ala343A |
| 331 | Cα | Tyr345A |
| 360 | Cα | Val374A |
| 382 | Cα | Asp396A |
| 385 | Cα | Leu399A |
| 406 | Cα | Met420A |
| 407 | Cα | Leu421A |
| 408 | C | Leu421A |

**Table S8. Active Site Residues of CatB Characterized by Molecular Dynamics Simulation**

| **Residue Index** | **Atom** | **Amino Acid** |
| --- | --- | --- |
| 46 | Cα | Cys122A |
| 206 | Cα | His282A |
| 226 | Cα | Asn302 |


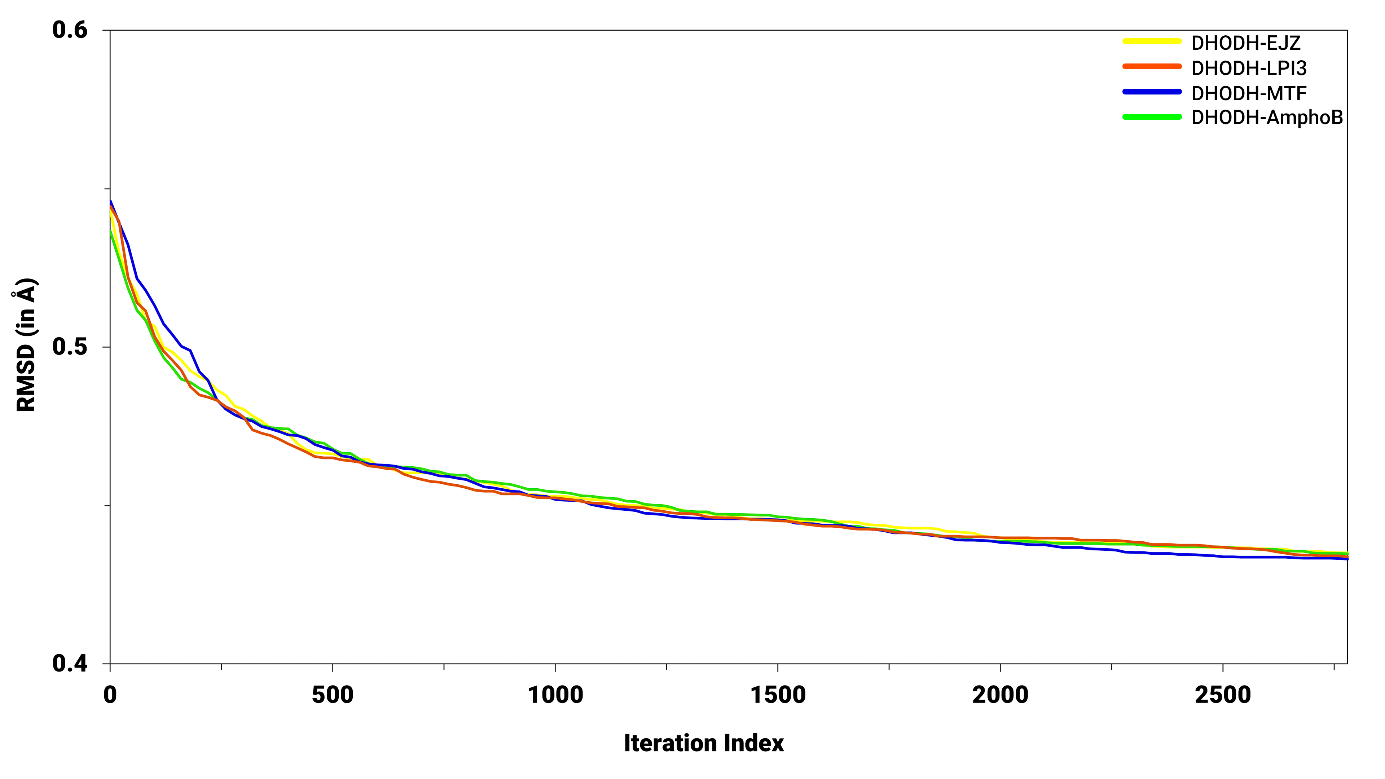


**Fig. S1** Convergence of the morphing trajectory for the complexes DHODH-EJZ (yellow line), DHODH-LPI3 (red line), DHODH-MTF (blue line) and DHODH-AmphoB (green line), indicating the RMSD variation of Ca (in Å) in relation to the iteration index.


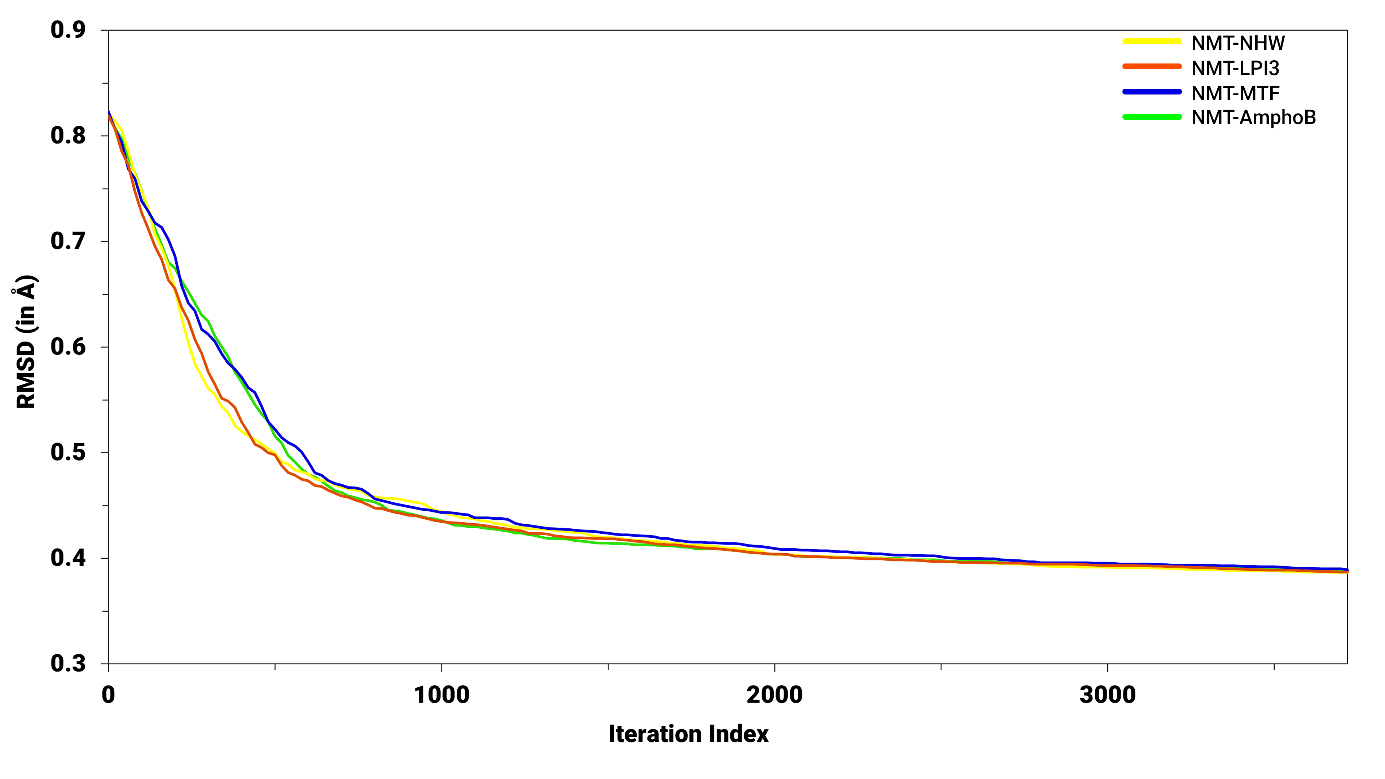


**Fig. S2** Convergence of the morphing trajectory for the complexes NMT-NHW (yellow line), NMT-LPI3 (red line), NMT-MTF (blue line) and NMT-AmphoB (green line), indicating the RMSD variation of Ca (in Å) in relation to the iteration index.


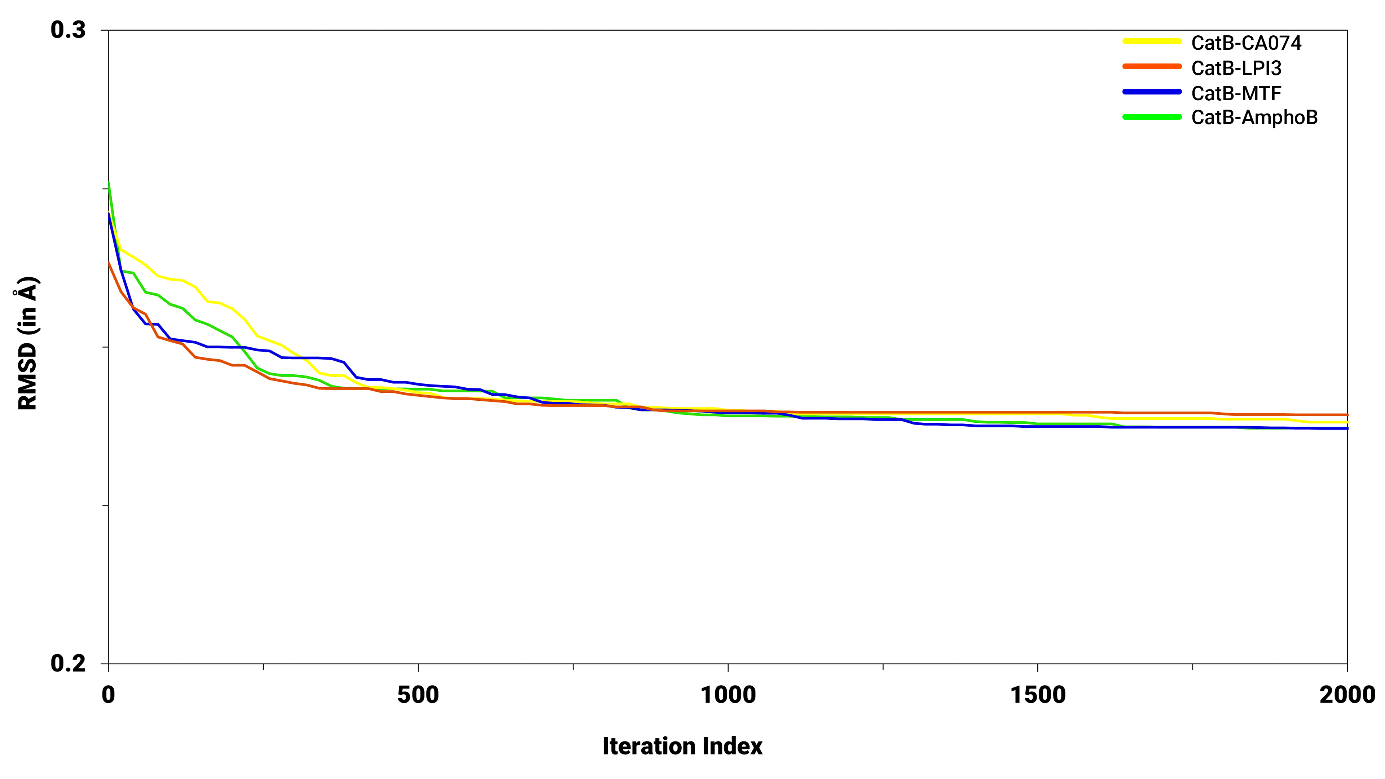


**Fig. S3** Convergence of the morphing trajectory for the complexes CatB-CA074 (yellow line), CatB-LPI3 (red line), CatB-MTF (blue line) and CatB-AmphoB (green line), indicating the RMSD variation of Ca (in Å) in relation to the iteration index.
